# Design Strategies for Compact Photoluminescent Liquid Crystals Based on Fluorinated Tolane Frameworks

**DOI:** 10.1002/tcr.202500346

**Published:** 2026-03-31

**Authors:** Shigeyuki Yamada, Motohiro Yasui, Tsutomu Konno

**Affiliations:** ^1^ Faculty of Molecular Chemistry and Engineering Kyoto Institute of Technology Kyoto Japan

**Keywords:** donor–π–acceptor architecture, fluorinated π‐conjugated materials, mesophase stabilization, molecular design strategy, photoluminescent liquid crystals

## Abstract

Fluorinated tolane derivatives offer rigid and compact π‐conjugated frameworks in which electronic structure and intermolecular interactions can be precisely modulated, making them attractive platforms for integrating mesomorphic order and photoluminescence within a single molecular architecture. In this account, we summarize our systematic efforts to establish molecular design strategies for compact fluorinated tolane‐based photoluminescent liquid crystals (PLLCs), with a particular focus on controlling molecular aggregation across crystalline and liquid–crystalline phases. Three complementary design approaches are discussed: molecular dimerization to reduce crystallinity and induce mesomorphism, partial fluorination combined with semifluoroalkoxy chains to balance electronic effects and aggregation moedes, and ionic functionalization using imidazolium termini to induce dynamic layered order and counteranion‐dependent control. Though these strategies, the roles of fluorination, spacer length, flexible chains, and ionic interactions in governing mesophase stability and solid‐state photoluminescence are elucidated. Collectively, these studies demonstrate how hierarchical control of molecular order enables the reconciliation of mesomorphic behavior and efficient emission within compact π‐conjugated systems, and they provide general design principles for the development of responsive emissive soft materials.

## Graphical abstract

This account highlights molecular design strategies for fluorinated tolane derivatives that integrate mesomorphic order with photoluminescence. The extent to which the fluorination pattern and flexible chain architecture govern the structure–property relationships to realize highly emissive and thermally tunable liquid–crystalline systems is elucidated.

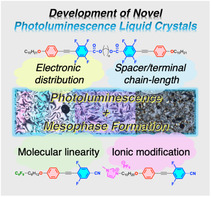

## 1 Introduction

The diphenylacetylene skeleton, commonly referred to as tolane, represents a linear and compact π‐conjugated framework that combines high structural rigidity with an extended π‐electron system [1, 2]. Owing to these features, tolane derivatives have attracted considerable attention as versatile building blocks for photofunctional materials [3, 4, 5, 6], including organic electronic wires [7, 8], nonlinear optical materials [9, 10, 11], and DNA‐photocleaving agents [12, 13]. At the same time, the rigid and rod‐like nature of the tolane core has rendered it an attractive mesogenic unit for the design of liquid–crystalline (LC) materials [14, 15, 16]. Despite these advantages, tolane frameworks have historically been regarded as unfavorable for emissive applications at ambient temperature. Conventional tolanes are typically nonemissive because the photoexcited linear ππ* state rapidly relaxes to a nonradiative *trans‐bent* πσ* excited state through internal conversion [17, 18, 19, 20]. In recent years, however, initial efforts to overcome this long‐standing limitation have been reported. Several studies have demonstrated that appropriate molecular design, such as tethering strategies [21, 22, 23, 24, 25], crystallization‐induced emission (CIE) [26, 27, 28, 29], aggregation‐induced enhanced emission (AIEE) [30, 31, 32], or the incorporation of donor–π–acceptor (D–π–A) architectures, can endow tolane derivatives with pronounced photoluminescence (PL) in the solid state [33, 34, 35, 36, 37, 38, 39, 40]. Against this background, our research has been guided by a central question: can a single, compact tolane‐based framework be rationally engineered to simultaneously exhibit liquid‐crystallinity and efficient solid‐state photoluminescence? Addressing this challenge requires not only the activation of emissions in tolane systems but also precise control over molecular aggregation and phase behavior. In this context, we have been focusing on fluorination as a key molecular design tool, thus exploiting its dual role in tuning both the electronic properties and intermolecular interactions of π‐conjugated systems.

**FIGURE 1:**
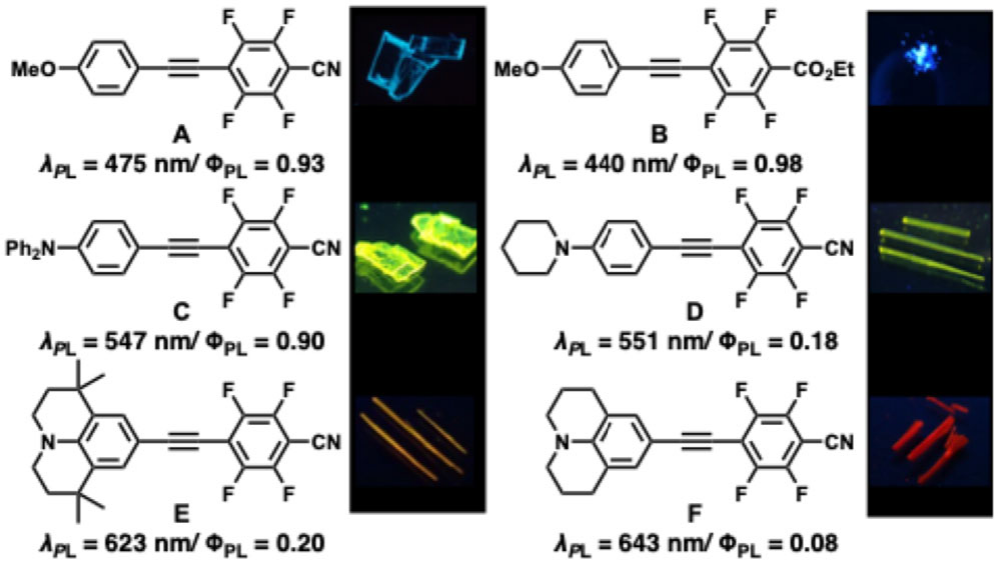
Solid‐state photoluminescent D–π–A tetrafluorinated tolane derivatives developed by our group, together with photographs of their solid‐state PL under UV irradiation.

In 2020, we reported a series of D–π–A‐type tetrafluorinated tolane derivatives [41, 42, 43, 44], in which an electron‐rich benzene ring bearing an alkoxy donor was connected to an electron‐deficient fluorinated aromatic ring via a C≡C bond. The PL of these compounds was strikingly enhanced upon crystallization: the PL quantum yield (Φ_PL_) in dilute solution (≈ 0.20) increased dramatically to values as high as 0.98 in the crystalline state. Building on this discovery, we subsequently developed a variety of solid‐state emissive tolane derivatives (**A**–**F**) [41, 45, 46, 47, 48], thereby achieving systematic color tuning from blue to red through precise modulation of the donor and acceptor units (Figure 1). These results clearly highlighted fluorination as an effective strategy for controlling the electronic density distribution and solid‐state emission behavior in compact π‐conjugated frameworks.

Notably, the introduction of a diphenylamino (NPh_2_) group as an electron‐donating unit afforded compound **C**, which displayed distinct crystalline polymorphs depending on the recrystallization conditions [45]. One polymorph exhibited yellow emission with a photoluminescence maximum (*λ*_PL_) at 563 nm and Φ_PL_ of 0.51. Such polymorphism‐dependent and mechanochromic PL behavior is well documented for organic emissive materials [49, 50, 51, 52, 53] and is generally attributed to changes in the intermolecular interactions arising from structural rearrangements within the crystal lattice. These observations underscore a key insight that guided our subsequent work: *the reversible modulation of intermolecular interactions provided a powerful strategy for switching the PL properties in the solid state.* These results highlighted the use of fluorination to be an effective strategy for controlling the electronic density distribution and solid‐state emission behavior within compact π‐conjugated frameworks. Notably, polymorphism‐dependent PL further underscores the importance of intermolecular interactions in governing the emissive properties in the solid state. Our earlier work was concerned with electronic density control in crystalline π‐systems [54], whereas the present study extends this concept to dynamic mesophases, in which molecular order and motion coexist.

**FIGURE 2:**
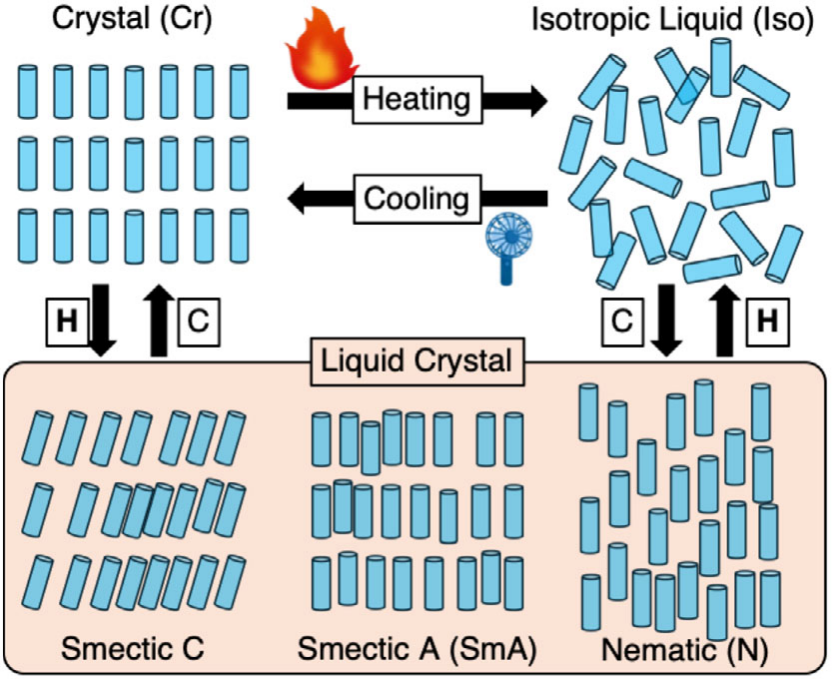
Schematic illustration of phase transitions and typical liquid–crystalline phases.

Phase transitions in molecular materials offer an attractive platform for reversible control. Upon heating, most organic compounds undergo a transition from an ordered crystalline (Cr) to an isotropic liquid (Iso) phase. In anisotropic rod‐like molecules composed of a rigid π‐conjugated core (mesogen) and flexible alkyl or alkoxy chains, one or more intermediate LC phase(s) often emerges between these two extremes (Figure 2) [55, 56, 57, 58]. LC phases uniquely combine long‐range orientational order with partial fluidity and are commonly classified as nematic (N) or smectic (Sm) phases, depending on the degree of positional order retained. Smectic phases can be further categorized into smectic A (SmA) or smectic C phases depending on the tilt angle between the molecular long axis and the layer normal (Figure 2).

From a photophysical perspective, LC phases represent an intermediate aggregation state in which molecular packing can be reversibly tuned without altering the chemical structure. Although numerous photoluminescent liquid crystals (PLLCs) have been reported [59, 60, 61, 62, 63, 64, 65, 66], most rely on molecular architectures in which the emissive and mesogenic units are distinct and connected by flexible linkers. These designs, which often result in bulky and synthetically complex molecules, limit their structural precision and practical applicability. Therefore, we sought to develop PLLCs with compact molecular architectures in which a single fluorinated tolane framework functions simultaneously as an emissive chromophore and a mesogenic core.

**FIGURE 3:**
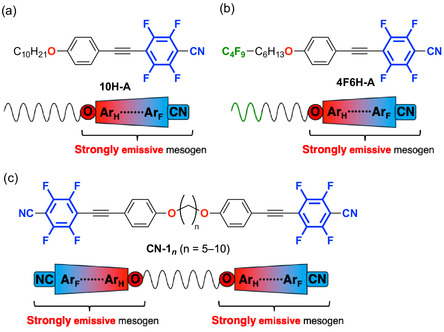
Chemical structures of (a) tetrafluorinated tolane **10H‐A** with a hydrocarbon chain and (b) tetrafluorinated tolane **4F6H‐A** with a semifluoroalkoxy chain, and (c) tetrafluorinated tolane‐based mesogenic dimers **CN‐1**_***n***_, as reported by our group. The colors in the figure represent the electron‐density distribution, where red indicates an electron‐rich region and blue indicates electron‐deficient regions.

As an initial attempt, flexible hydrocarbon or semifluoroalkoxy chains were introduced into D–π–A‐type tetrafluorinated tolane derivatives, exemplified by compounds **10H‐A** and **4F6H‐A** (Figure 3a, b) [67]. Although these molecules retained strong solid‐state PL, they were highly crystalline and failed to form LC phases. To overcome this limitation, we employed molecular dimerization as a strategy to reduce the crystallinity without sacrificing emission efficiency. In mesogenic dimer **CN‐1**_***n***_, two tetrafluorinated tolane units were linked using a flexible alkylene‐1,*n*‐dioxy spacer (Figure 3c) [68]. This design successfully induced LC behavior, although the mesophase stability was highly sensitive to the spacer length.

**FIGURE 4:**
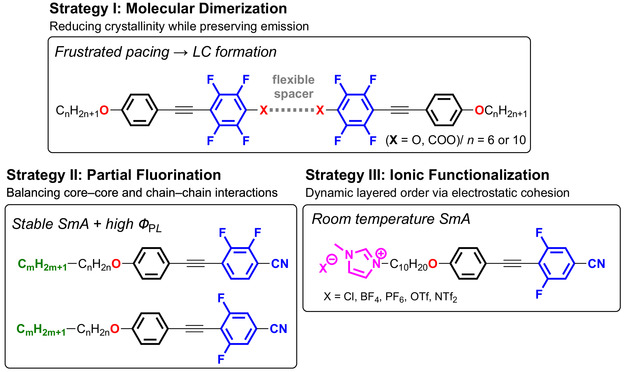
Conceptual overview of the three complementary molecular design strategies to develop compact photoluminescent liquid crystals based on a fluorinated tolane framework.

These studies revealed a delicate balance between the mesomorphic order and PL efficiency, and motivated the exploration of additional molecular design strategies capable of reconciling these competing requirements. In this account, we summarize our subsequent efforts based on three complementary design concepts (Figure 4).

(i) Mesogenic dimerization to modulate crystallinity and phase behavior [69, 70, 71],

(ii) Partial fluorination combined with semifluoroalkoxy chains to fine‐tune intermolecular interactions [72], and

(iii) Ionic functionalization using imidazolium termini to introduce dynamic ordering effects [73].

By Systematically examining the influence of fluorination patterns, spacer lengths, flexible chains, and ionic groups on the phase transitions and PL, we aimed to extract general design principles for compact, fluorinated, tolane‐based PLLCs. Detailed synthetic procedures and full characterization data for all new compounds are provided in the Supporting Information.

## 2 Molecular Dimerization as a Strategy to Balance Mesomorphic Order and Photoluminescence

Highly fluorinated tolane derivatives exhibit excellent solid‐state PL owing to their rigid π‐conjugated frameworks and strong intermolecular interactions. However, these features often lead to excessive crystallinity, which suppresses the formation of LC phases. To mitigate this intrinsic trade‐off between crystallinity and emission, we explored molecular dimerization as a design strategy to modulate the molecular packing while preserving the favorable electronic structure of the tolane‐based emissive core. The connection of two photoluminescent tolane mesogenic units via a flexible spacer would enable molecular mobility and packing frustration to be introduced without fundamentally altering the p‐conjugated framework. This approach was expected to reduce the crystallinity, promote mesophase formation, and enable the coexistence of mesomorphic order and solid‐state PL.

### 2.1 Concept and Design Rationale

Molecular dimerization is employed as the central motivation to overcome the strong crystallinity inherent to highly fluorinated tolane monomers. Despite the high Φ_PL_ of these monomers in the crystalline state, their rigid and planar molecular structures tend to favor close‐packed crystalline lattices, which tend to prevent the emergence of LC phases. The introduction of a flexible spacer between two identical mesogenic units provides an effective means of weakening the long‐range crystalline order, while retaining the electronic characteristics responsible for efficient emission. In this context, dimerization was not intended to enhance the emission directly but rather to decouple the mesomorphism from the emissive efficiency, which allows structural parameters such as the spacer length, terminal chains, and fluorination patterns to be independently tuned. This concept underpins all dimeric systems discussed in this section.

### 2.2 Emergence and Limitation of Mesomorphism in Tolane Dimers

**FIGURE 5:**
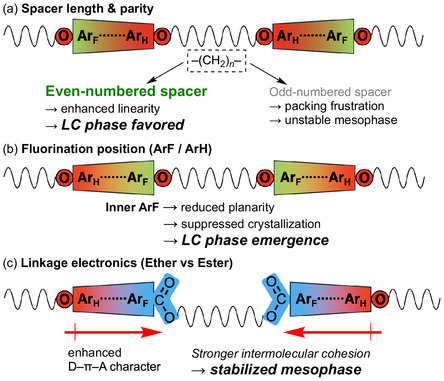
Schematic illustration of the structural and electronic parameters governing mesophase stability in fluorinated tolane‐based mesogenic dimers. (a) Effect of spacer length and parity on molecular linearity and mesophase formation. (b) Influence of the relative positions of fluorinated and nonfluorinated aromatic rings on molecular planarity and crystallization tendency. (c) Electronic modulation of the mesogenic core by replacing ether linkages with ester units to enhance the donor–π–acceptor character and intermolecular cohesion. The colors in the figure represent the electron‐density distribution, where red indicates electron‐rich regions, blue indicates electron‐deficient regions, and green indicates areas with intermediate electron density.

As an initial demonstration of this concept, we previously reported a series of mesogenic dimers (**CN‐1**_***n***_), in which tetrafluorinated tolane units (**A**) with a high crystalline‐state Φ_PL_ of ≈0.9 were connected via flexible alkylene‐1,*n*‐dioxy spacers (*n* = 5–10) [68]. These dimers displayed solid‐state PL with Φ_PL_ values ranging from 0.26 to 0.72, confirming that dimerization did not compromise the emissive nature of the tolane core. However, thermal analyses revealed that LC phases appeared only for derivatives with longer spacers (*n* = 8–10), and even in these cases, the temperature range of the mesophase was extremely narrow, of the order of 6°C (Figure 5a).

These findings clearly demonstrate that molecular dimerization is effective for inducing mesomorphism in highly emissive tolane systems but also highlight the inherent limitations of this simple design with respect to mesophase stability. Thus, the **CN‐1**_***n***_ series served as a proof‐of‐concept to establish dimerization as a viable strategy while simultaneously revealing the need for more refined control over the molecular geometry and intermolecular interactions.

### 2.3 Structural Parameters Governing Mesophase Stability

To address the limited LC stability observed in the **CN‐1**_***n***_ series, we systematically investigated the effects of key structural parameters, namely the spacer length, terminal chain structure, and position of fluorine substituents, on mesophase formation and stability.

#### 2.3.1 Spacer Length and Terminal Chain Effects

**FIGURE 6:**
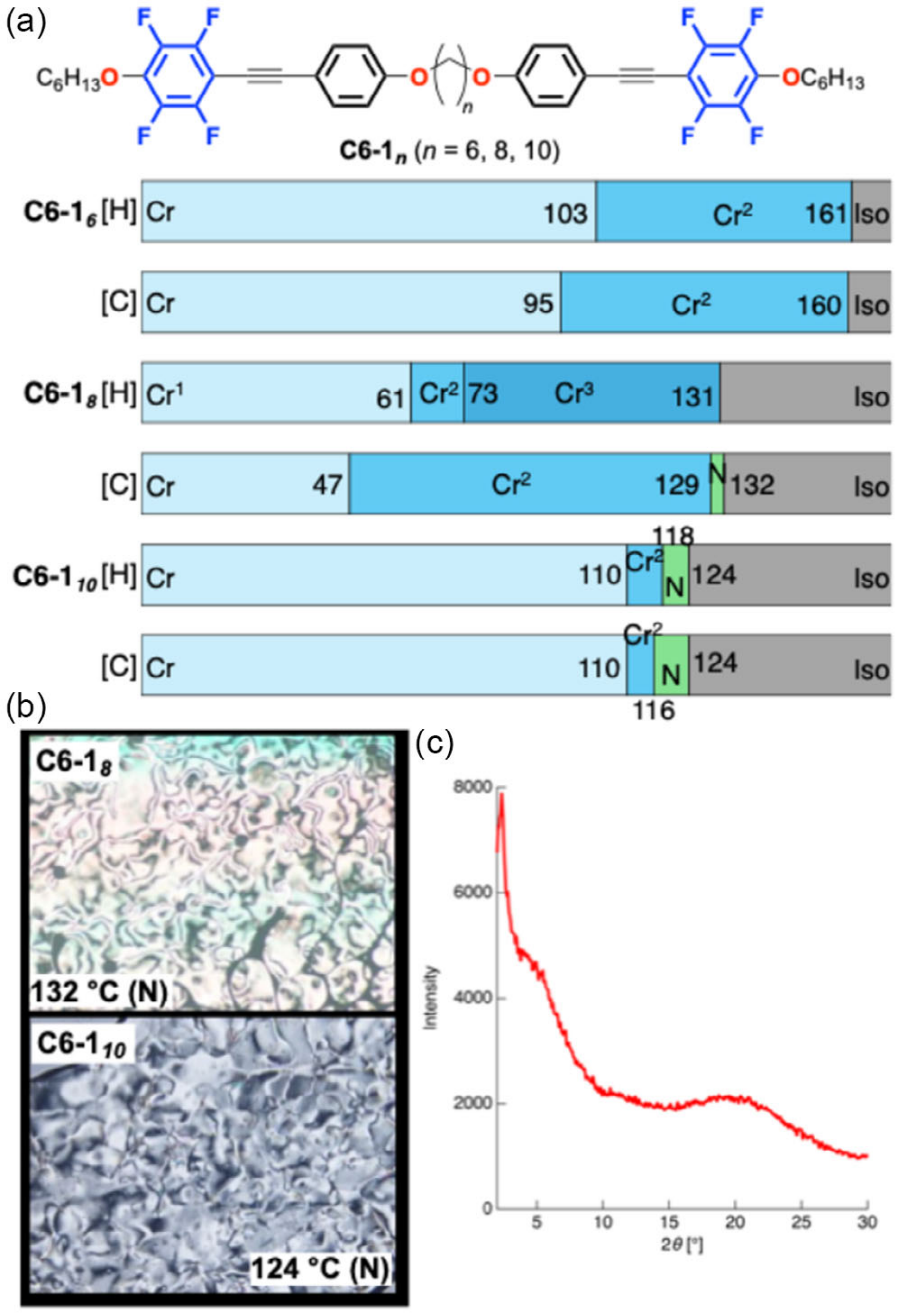
(a) Chemical structures of the **C6‐1**_***n***_ series (*n* = 6, 8, 10) and DSC results recorded under nitrogen (N_2_) at a scan rate of 5.0°C min^−1^ to determine their phase transitions. (b) Polarized optical microscopy (POM) textures observed in the mesophase temperature range. (c) VT‐PXRD pattern of the **C6‐1**_***10***_ in N phase at 120°C during the cooling process.

The phase transition behavior of the **1**_***n***_ series, in which both of the two outermost aromatic rings in the mesogenic core are fluorinated, was first examined [69]. Compounds **C6‐1**_***n***_ (*n* = 6, 8, and 10) bearing hexyloxy (C_6_H_13_O) terminal chains were synthesized and analyzed by differential scanning calorimetry (DSC) and polarized optical microscopy (POM). DSC measurements were conducted over three consecutive heating–cooling cycles to confirm the reproducibility of the phase‐transition behavior (Figure 6).

Compound **C6‐1**_***6***_ exhibited only a direct crystalline (Cr)‐to‐isotropic (Iso) phase transition. In contrast, **C6‐1**_***8***_ and **C6‐1**_***10***_ exhibit mesophases between the Cr and Iso phases. POM observations revealed typical four‐brushed Schlieren textures, while variable temperature‐powder X‐ray diffraction (VT‐PXRD) patterns showed only a broad halo‐peak around 2*θ* ≈ 20°, indicative of the absence of positional order. These results identify the mesophases as N phases. Although the clearing temperature (*T*_c_) decreased systematically with increasing spacer length owing to enhanced molecular flexibility, the temperature ranges of the N phase were extremely narrow: only 3°C for **C6‐1**_***8***_ and 8°C for **C6‐1**_***10***_.

**FIGURE 7:**
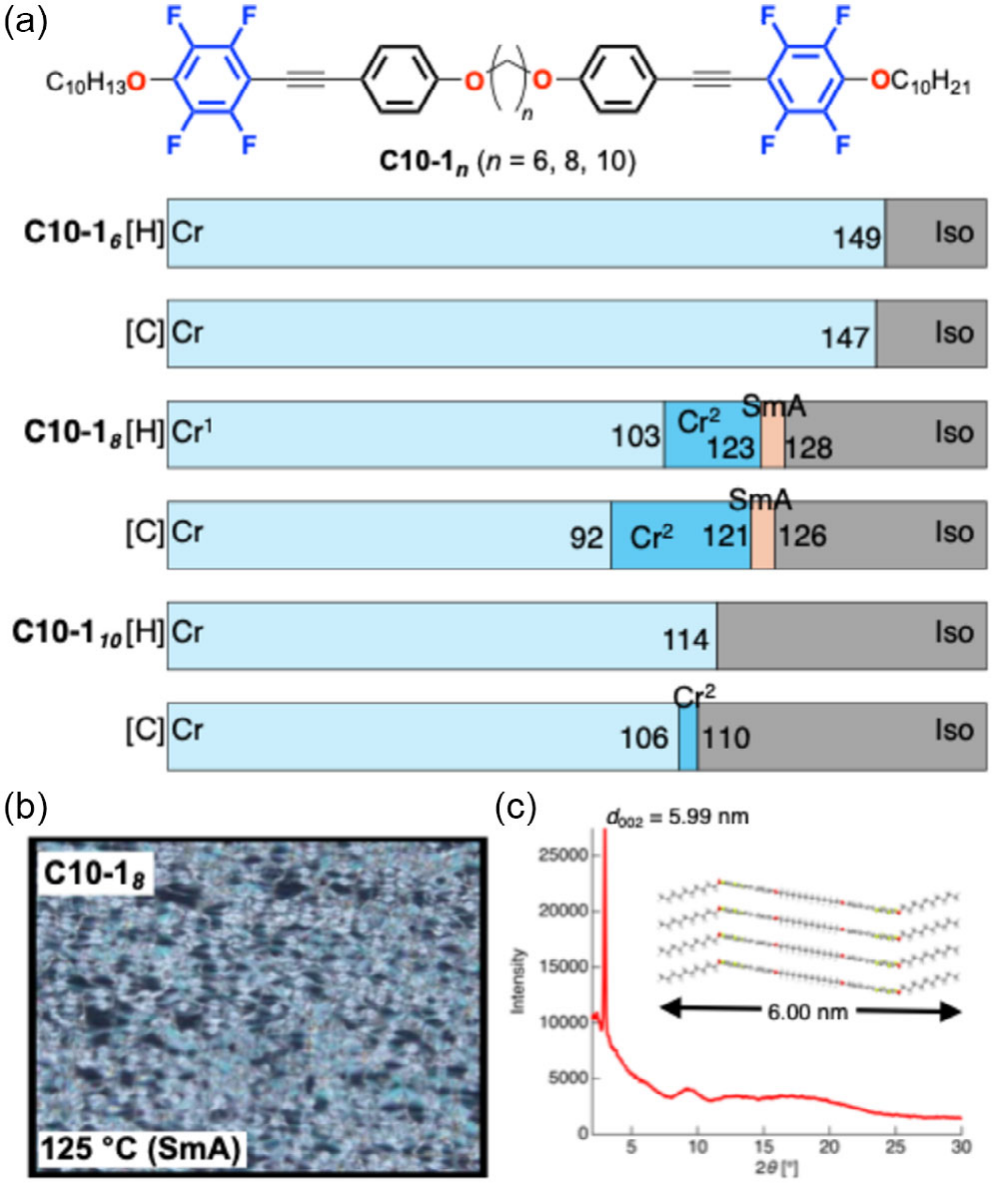
(a) Chemical structures of the **C10‐1**_***n***_ series (*n* = 6, 8, 10) and DSC results recorded under N_2_ at a scan rate of 5.0°C min^−1^ to determine their phase transitions. (b) POM textures observed in the mesophase temperature range. (c) VT‐PXRD pattern of the **C10‐1**_***8***_ in SmA phase at 125°C during the cooling process.

The terminal chains were extended to further stabilize the mesophase. A series of **C10‐1**_***n***_ (*n* = 6, 8, and 10) derivatives bearing decyloxy (C_10_H_21_O) terminal chains were synthesized and examined (Figure 7) [71].

The compound **C10‐1**_***6***_ again exhibited only a direct Cr → Iso phase transition, whereas **C10‐1**_***8***_ displayed a smectic A (SmA) phase over a narrow temperature range of ≈ 5°C. VT‐PXRD revealed a layered *d*‐spacing (*d* = 6.00 nm) comparable to the molecular length, thus confirming the formation of the SmA phase. Notably, further elongation of the spacer (**C10‐1**_***10***_) suppressed mesophase formation, indicating that the spacer and terminal chain lengths exert competing influences on the crystallinity and layered ordering.

#### 2.3.2 Position of Fluorine Substituents and Molecular Geometry

**FIGURE 8:**
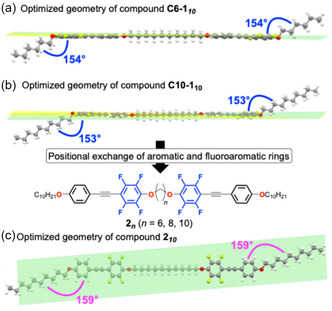
Optimized geometries of (a) **C6‐1**_***10***_, (b) **C10‐1**_***10***_, and (c) **2**_***n***_, obtained from DFT calculations at the M06‐2X/6‐31+G(d, p)//M06‐2X/6‐31G(d) level of theory.

To rationalize the restricted appearance of LC phases in the **C6‐1**_***n***_ and **C10‐1**_***n***_ series, density functional theory (DFT) calculations [74, 75, 76, 77] were performed to determine the optimized molecular geometries (Figure 8). In compounds **C6‐1**_***10***_ and **C10‐1**_***10***_ (Figure 8a, b), the two mesogenic units and spacer adopted a nearly coplanar conformation, whereas the terminal flexible chains deviated significantly from this plane by ≈ 153°–154°. This highly planar molecular geometry is expected to stabilize the Cr phase, thereby suppressing mesophase formation.

**FIGURE 9:**
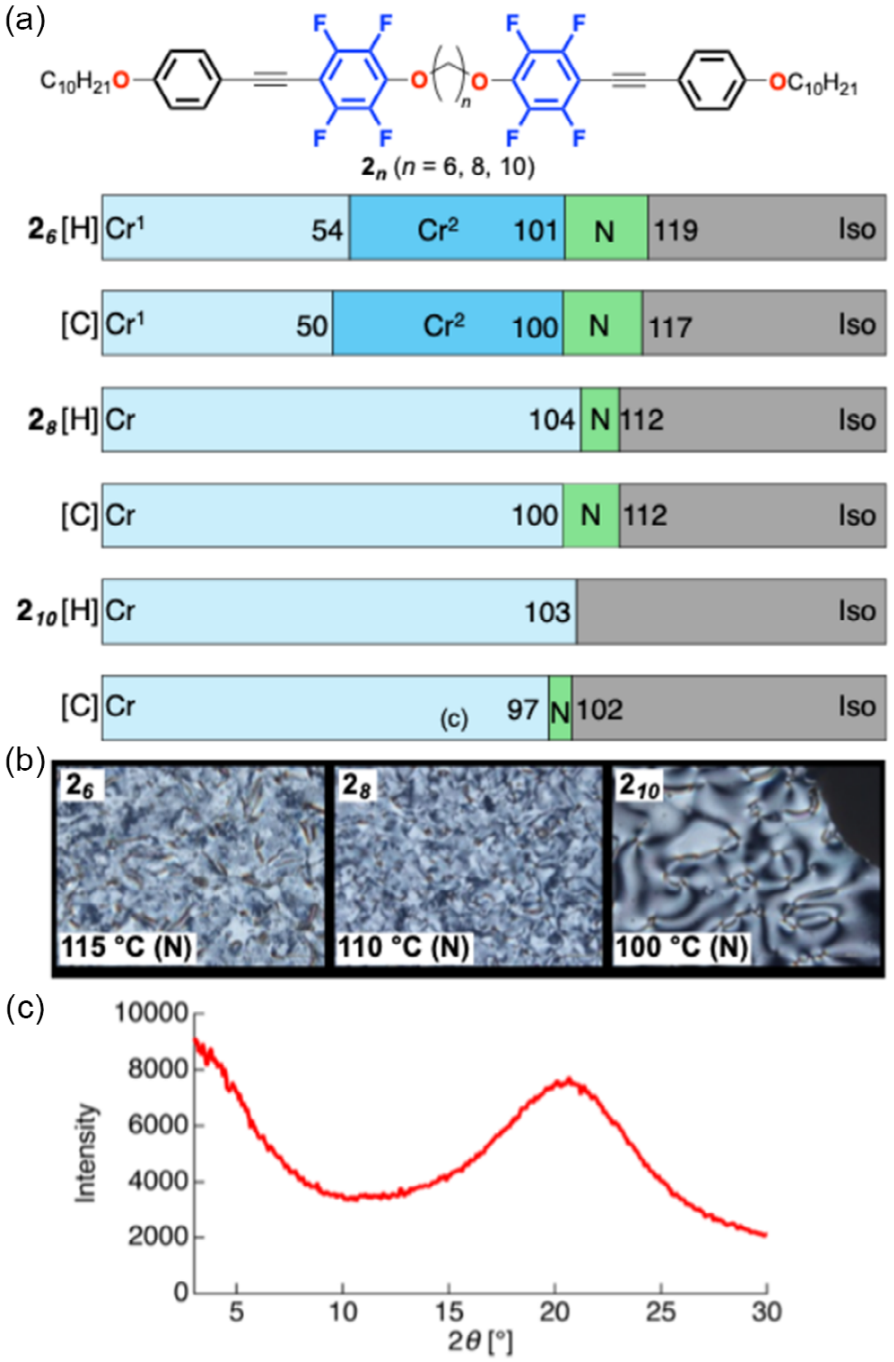
(a) Chemical structures of the **2**_***n***_ series (*n* = 6, 8, 10) and DSC results recorded under N_2_ at a scan rate of 5.0°C min^−1^ to determine their phase transitions. (b) POM textures observed in the mesophase temperature range. (c) VT‐PXRD pattern of the **2**_***8***_ in the N phase at 109°C during the cooling process.

Attempting to reduce the crystallinity, the fluorinated aromatic rings were exchanged with the nonfluorinated aromatic rings such that the fluorinated rings occupied the innermost positions in the mesogenic core. In the resulting compound **2**_***10***_, the π‐conjugated plane of the mesogenic unit was oriented perpendicular to the spacer, yielding a more linear molecular shape with a reduced tendency toward crystallization (Figure 8c). Based on this rationale, a new series of compounds, **2**_***n***_ (*n* = 6, 8, and 10), with their fluorinated aromatic rings in the innermost positions, were synthesized and evaluated (Figure 9) [70].

As anticipated, **2**_***6***_ exhibited a mesophase during both heating and cooling. POM observations revealed Schlieren textures, and VT‐PXRD displayed a broad halo peak characteristic of the N phase. The mesophase temperature range, however, decreased systematically with increasing spacer length: 17–18°C for **2**_***6***_, to 8–12°C for **2**_***8***_, and 5°C for **2**_***10***_, indicating that although repositioning of the fluorinated aromatic ring effectively promoted mesophase formation, excessive spacer flexibility remained detrimental to LC stability.

### 2.4 Electronic Modulation of Mesogenic Cores

**FIGURE 10:**
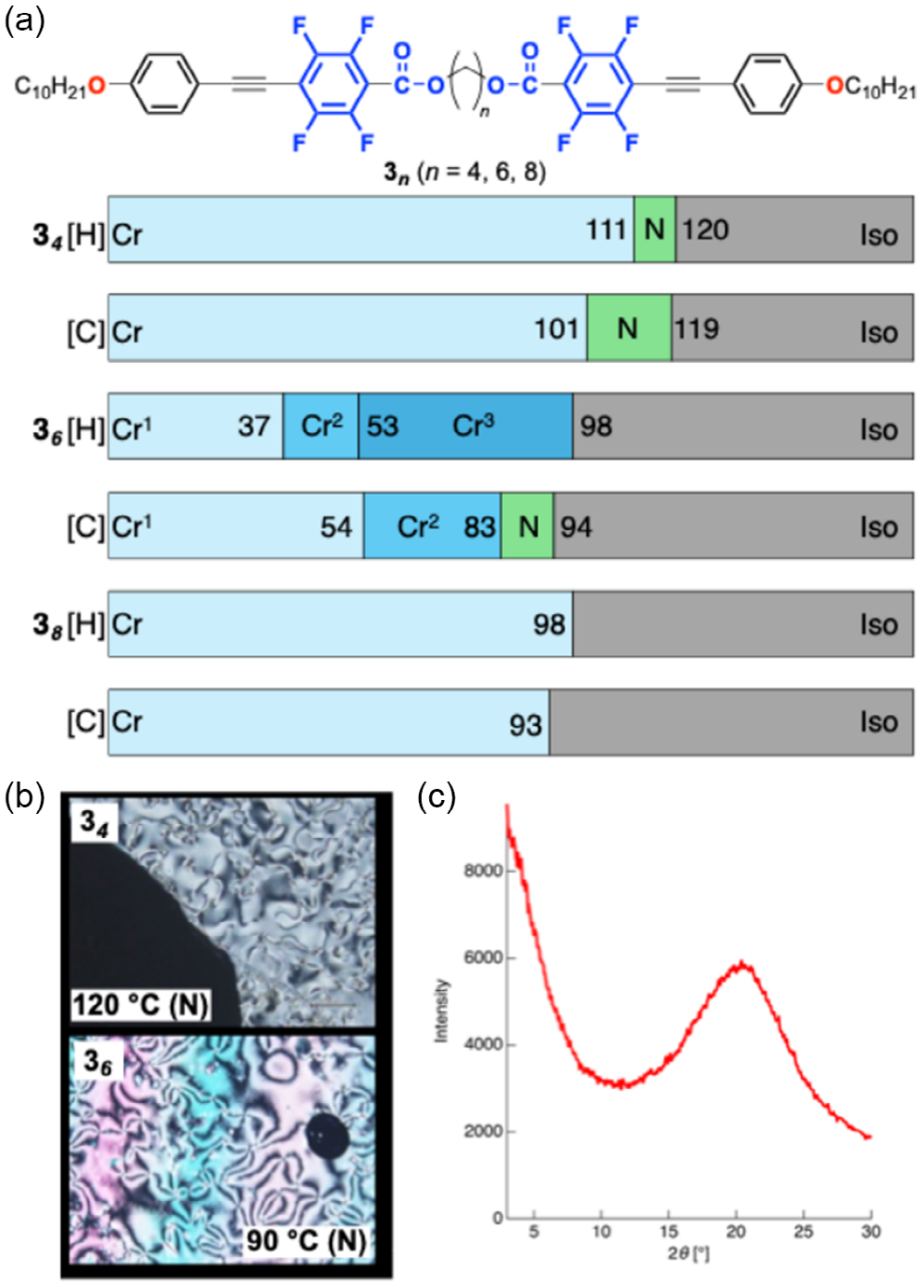
(a) Chemical structures of the **3**_***n***_ series (*n* = 4, 6, 8) from DSC results recorded under N_2_ at a scan rate of 5.0°C min^−1^ to determine their phase transitions. (b) POM textures observed in the mesophase temperature range. (c) VT‐PXRD pattern of the **3**_***6***_ in N phase at 92°C during the cooling process.

In both the **1**_***n***_ and **2**_***n***_ series, the ether linkage between the mesogenic core and the spacer acts as an electron‐donating group, resulting in a relatively uniform electron distribution across the π‐conjugated framework. This electronic feature likely weakens the intermolecular interactions between the mesogenic units to limit the mesophase stability. To enhance intermolecular cohesion, the ether linkage was replaced with an electron‐withdrawing ester unit, thereby reinforcing the D–π–A character of the mesogenic core. Based on this design strategy, a third series of dimers, **3**_***n***_ (*n* = 4, 6, and 8), was synthesized and investigated (Figure 10) [71].

Compound **3**_***6***_ exhibited monotropic LC behavior with a stable mesophase over ≈11°C, whereas the LC behavior of compound **3**_***4***_ was enantiotropic with mesophase temperature ranges of 9°C upon heating and 18°C upon cooling. In contrast, elongation of the spacer, as in compound **3**_***8***_, destabilized the LC phase, which is consistent with the trends observed for the **2**_***n***_ series. All the mesophases were identified as N phases based on their Schlieren textures and the absence of sharp reflections in the VT‐PXRD patterns. Notably, the **3**_***n***_ series also exhibited a distinct odd–even effect, wherein compounds with even‐numbered spacers formed N phases whereas those with odd‐numbered spacers formed SmA phases**.**

### 2.5 Photoluminescence Across Crystalline and Mesophases

**FIGURE 11:**
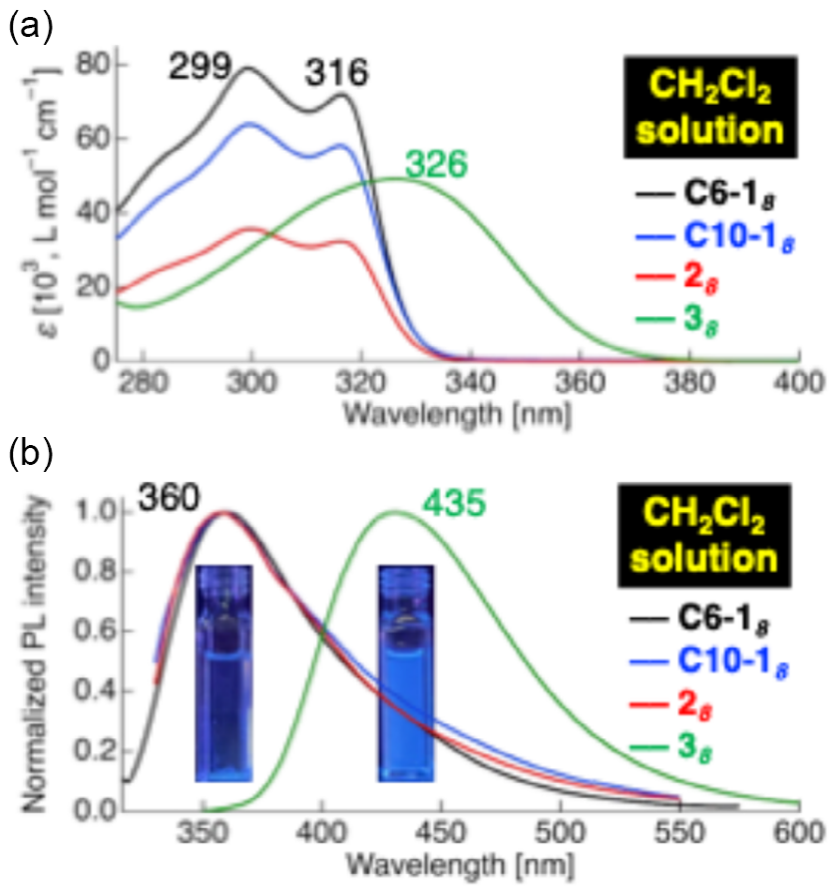
(a) UV–vis absorption and (b) PL spectra of **C6‐1**_***8***_, **C10‐1**_***8***_, **2**_***8***_, and **3**_***8***_ in CH_2_Cl_2_ solution, shown as representative examples. Concentrations: 1.0 × 10^−5^ mol L^−1^ for absorption and 1.0 × 10^−6^ mol L^−1^ for PL measurements.

**FIGURE 12:**
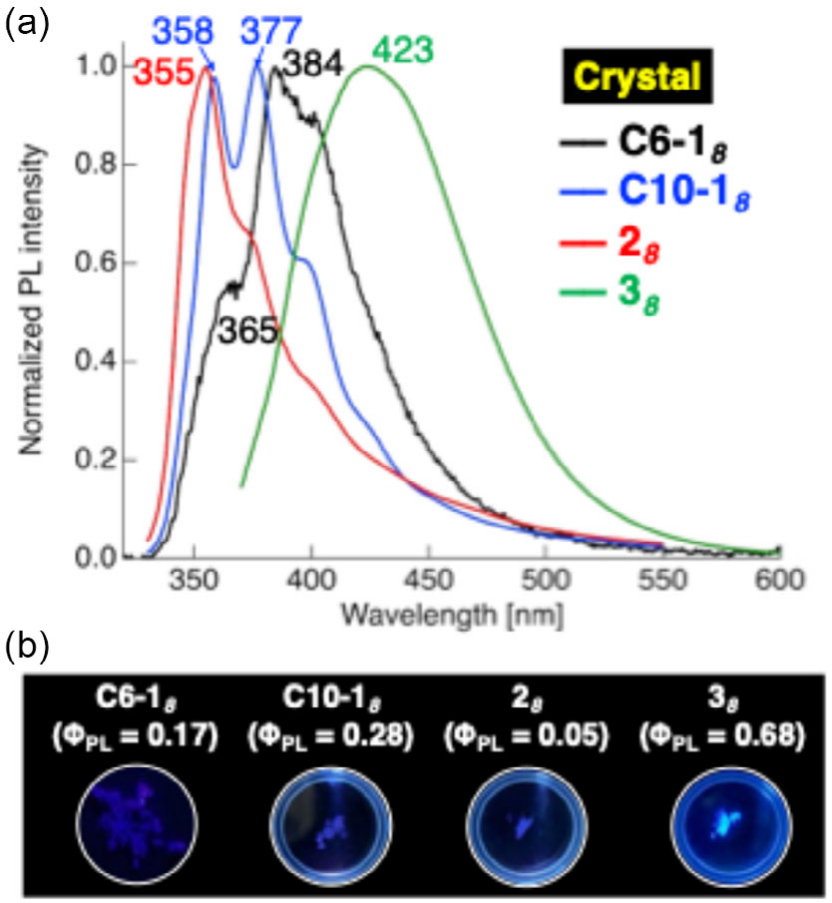
(a) PL spectra of **C6‐1**_***8***_ (*λ*_ex_ = 300 nm), **C10‐1**_***8***_ (*λ*_ex_ = 300 nm), **2**_***8***_ (*λ*_ex_ = 295 nm), and **3**_***8***_ (*λ*_ex_ = 347 nm) in the crystalline state. (b) Corresponding photographs of their solid‐state photoluminescence under UV irradiation.

The photophysical properties of the tetrafluorinated‐tolane‐based mesogenic dimers were investigated in a dilute CH_2_Cl_2_ solution and in the Cr state (Table 1). Representative absorption and PL spectra of the compounds bearing octylene spacers, viz., **C6‐1**_***8***_, **C10‐1**_***8***_, **2**_***8***_, and **3**_***8***_, are shown in Figures 11 and 12.

**TABLE 1:** Photophysical properties of the **C6‐1**_***n***_, **C10‐1**_***n***_, **2**_***n***_, and **3**_***n***_ series in CH_2_Cl_2_ solution and in the crystalline state.

	Solution			Crystal	
Compound	*λ*_abs_ [nm] (*ε* [10^3^, L mol^–1^ cm^–1^])^a^	*λ*_PL_ [nm]^b^	Φ_PL_ c	*λ*_PL_ [nm]	Φ_PL_ c
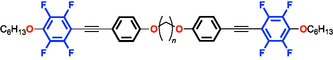					
**C6‐1**_***6***_	299 (46.4), 316 (42.5)	360	0.03	361, 378	0.12
**C6‐1**_***8***_	299 (79.2), 316 (72.0)	360	0.01	365, 384, 401	0.17
**C6‐1**_***10***_	299 (56.5), 317 (51.8)	360	0.03	363, 378, 398*sh*	0.19
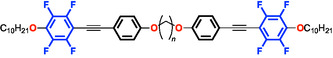					
**C10‐1**_***6***_	300 (80.6), 316 (74.0)	358	0.03	358	0.14
**C10‐1**_***8***_	300 (63.9), 316 (58.3)	359	0.03	358, 377, 396*sh*	0.28
**C10‐1**_***10***_	299 (64.9), 316 (58.7)	358, 418sh	0.04	360, 376, 398*sh*	0.14
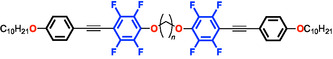					
**2**_***6***_	300 (24.7), 317 (24.0)	360, 384sh	0.03	355, 372*sh*	0.11
**2**_***8***_	300 (35.8), 316 (32.4)	358	0.03	355, 372*sh*	0.051
**2**_***10***_	300 (61.9), 316 (55.7)	357	0.03	372	0.091
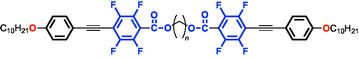					
**3**_***4***_	327 (35.2)	437	0.41	399, 421, 449	0.64
**3**_***6***_	326 (44.5)	435	0.40	400, 420sh, 449*sh*	0.68
**3**_***8***_	326 (49.3)	430	0.39	423	0.77

a Concentration: 1.0 × 10^–5^ mol L^–1^;

b Concentration: 1.0 × 10^–6^ mol L^–1^;

c Determined using an absolute photoluminescence quantum yield measurement system with an integrating sphere. *sh.* = shoulder peak.

Compounds **C6‐1**_***8***_, **C10‐1**_***8***_, and **2**_***8***_ exhibited absorption maxima (*λ*_abs_) at ≈299 nm and 316 nm, whereas compound **3**_***8***_ displayed a red‐shifted absorption band at 326 nm due to the enhanced D–π–A character imparted by the ester linkage. Time‐dependent density functional theory (TD‐DFT) calculations confirmed that these absorptions corresponded to the transitions from the highest occupied molecular orbital (HOMO)/HOMO–1 to the lowest unoccupied molecular orbital (LUMO)/LUMO + 1, with compound **3**_**8**_ exhibiting a smaller HOMO–LUMO energy gap (ΔE_H–L_).

Upon excitation, the dioxy‐linked compounds **C6‐1**_***8***_, **C10‐1**_***8***_, and **2**_***8***_ emitted near‐ultraviolet (UV) fluorescence in solution with relatively low Φ_PL_ values, whereas the fluorescence efficiencies of the ester‐linked **3**_***n***_ series were significantly enhanced. In the Cr state, the PL of all series intensified relative to that in solution, with the 3n series being strong blue emitters with high Φ_PL_ values of 0.64–0.77.

**FIGURE 13:**
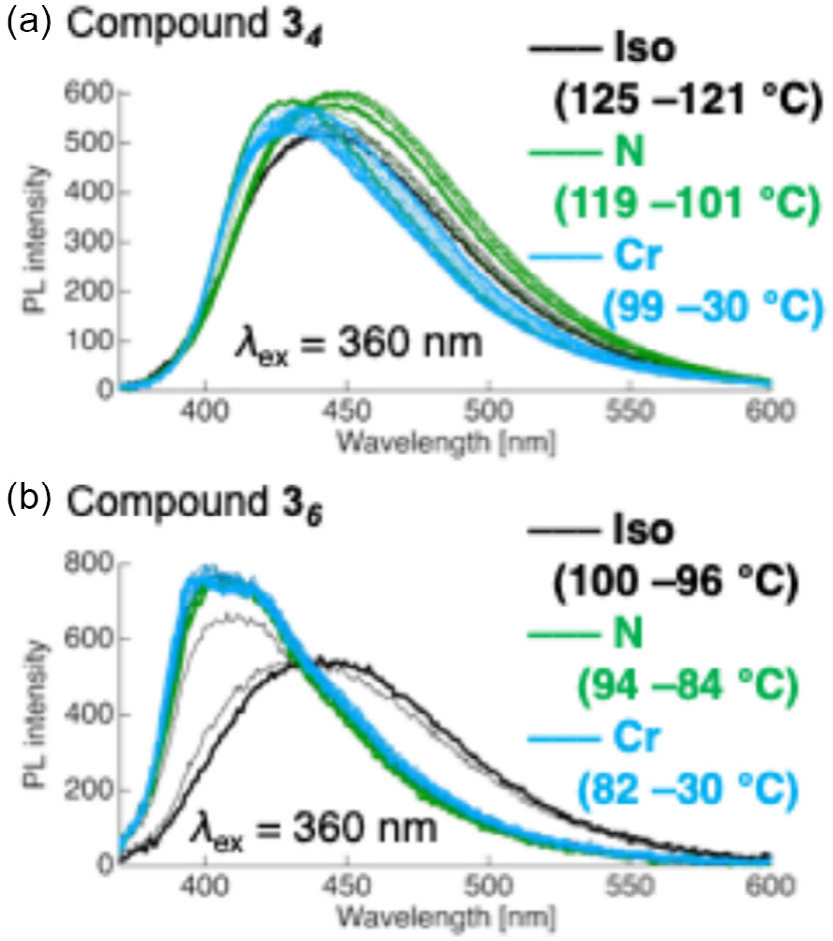
Evolution of PL behavior of compounds (a) **3**_***4***_ and (b) **3**_***6***_ during the Iso → N → Cr phase transitions upon cooling.

The temperature‐dependent PL behaviors of compounds **3**_***4***_ and **3**_***6***_ further demonstrated multistate emission across the Iso, LC, and Cr phases (Figure 13). Gradual spectral shifts and changes in Φ_PL_ accompanied the phase transitions, reflecting the reversible modulation of intermolecular interactions during the cooling process.

### 2.6 Design Insights from Dimeric Tolane Systems

Taken together, these results establish molecular dimerization as an effective strategy for mitigating the trade‐off between crystallinity and PL in fluorinated tolane‐based systems. The spacer length, terminal chain structure, and position of the fluorine substituents in the mesogenic core were found to be critical and interdependent parameters that govern the mesophase stability and emission efficiency. Excessive molecular planarity or flexibility suppresses the LC behavior, whereas appropriate geometric and electronic modulation enables the coexistence of mesomorphic order and strong solid‐state PL.

## 3 Balancing Mesophase Formation and Photoluminescence Through Partial Fluorination

In Section 2, molecular dimetrization was shown to be an effective strategy for inducing mesomorphism in highly emissive fluorinated tolane systems. However, even in dimeric architectures, excessive fluorination of the mesogenic core often leads to high crystallinity, which limits the stability of the LC phases. These observations prompted us to explore an alternative and complementary design strategy based on partial fluorination, in which the degree and distribution of fluorine atoms were carefully balanced between the mesogenic core and flexible chains.

### 3.1 Design Hypothesis: Fluorination Balance Between Core and Flexible Chains

**FIGURE 14:**
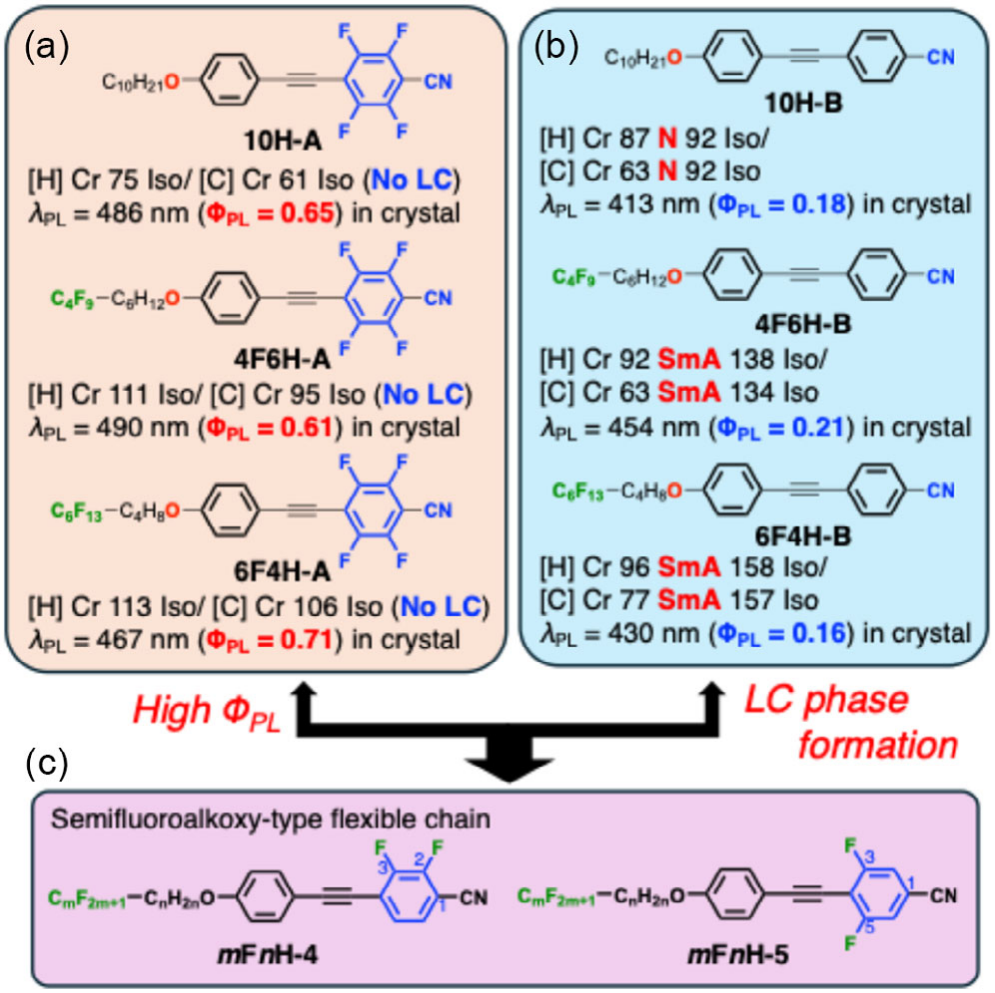
Chemical structures, phase‐transition behaviors, and PL properties of (a) tetrafluorinated and (b) nonfluorinated tolane derivatives previously developed by our group, together with (c) the chemical structure of the difluorinated tolane derivatives bearing a semifluoroalkoxy chain proposed in this study.

As discussed above, the tetrafluorinated tolane derivatives bearing hydrocarbon or semifluoroalkoxy chains, such as **10H‐A**, **4F6H‐A**, and **6F4H‐A**, exhibited high Φ_PL_ in the Cr state [18]. Nevertheless, owing to their high crystallinities, none of these compounds formed LC phases (Figure 14a). In contrast, the corresponding nonfluorinated tolane derivatives, viz., **10H‐B**, **4F6H‐B**, and **6F4H‐B**, readily formed N or SmA phases, but their Φ_PL_ values decreased markedly to ≈ 0.21 due to their weakened intermolecular interactions (Figure 14b) [78].

These contrasting behaviors suggested that excessive fluorination stabilizes the Cr phase at the expense of mesophase formation, whereas insufficient fluorination compromises the emission efficiency in the solid state. Therefore, we hypothesized that a regime of intermediate fluorination, achieved by the partial fluorination of the tolane core combined with semifluoroalkoxy flexible chains, could provide an optimal balance between mesomorphic ordering and PL behavior.

### 3.2 Semifluoroalkoxy Chains as Mesophase‐Stabilizing Elements

**FIGURE 15:**
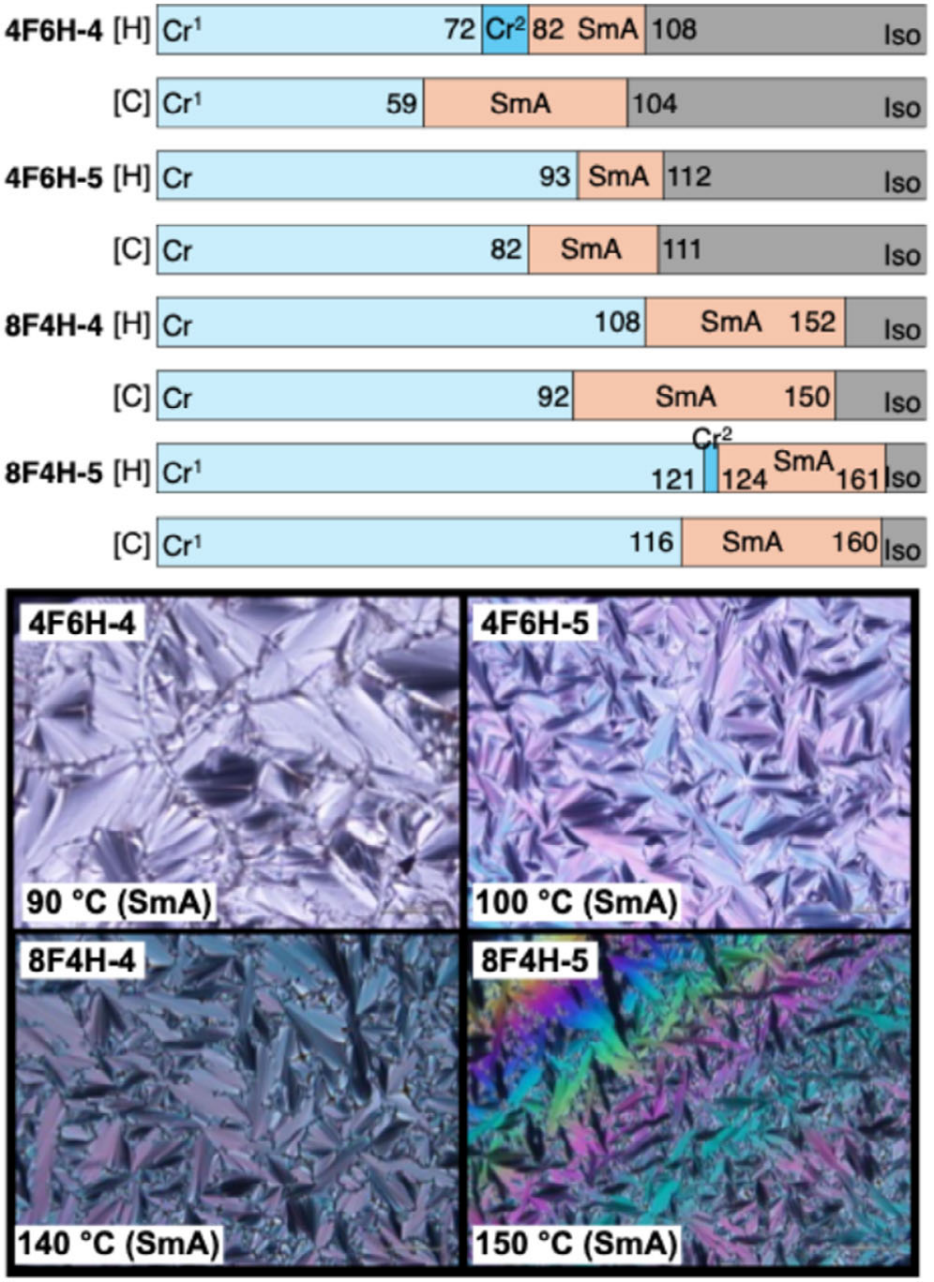
Phase‐transition behaviors of **4F6H‐4**, **4F6H‐5**, **8F4H‐4**, and **8F4H‐5** based on DSC results recorded under N_2_ at a scan rate of 5.0°C min^−1^, along with their POM textures observed in the mesophase.

To test this hypothesis, we designed and synthesized difluorinated tolane derivatives bearing semifluoroalkoxy flexible chains, which are known to promote the formation of layered mesophases (Figure 14c) [72]. Two series of compounds were prepared: Series **4** featuring fluorine substituents at the 2,3‐positions of the electron‐deficient aromatic ring and Series **5** featuring fluorine substituents at the 3,5‐positions. Compounds bearing nonafluorodecyloxy chains, **4F6H‐4** and **4F6H‐5**, and heptadecafluorododecyloxy chains, **8F4H‐4** and **8F4H‐5**, were synthesized and purified. DSC and POM analyses revealed that all these difluorinated derivatives exhibited enantiotropic LC behavior by forming mesophases upon heating and cooling (Figure 15).

POM observations of both compounds revealed characteristic fan‐shaped textures that transformed into dark domains when applying shear stress, indicating SmA phases.

**FIGURE 16:**
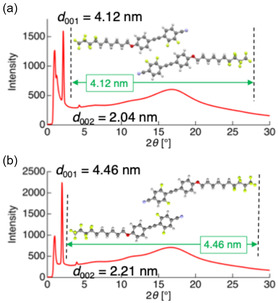
Powder X‐ray diffraction (PXRD) patterns of (a) **4F6H‐4** acquired at 85°C and (b) **4F6H‐5** at 100°C during the cooling process.

VT‐PXRD measurements at mesophase temperatures (85°C for **4F6H‐4** and 100°C for **4F6H‐5**) further confirmed the formation of SmA phases through the appearance of distinct (001) and (002) reflections (Figure 16). The calculated layer spacing exceeds the molecular lengths estimated by DFT, indicating the formation of interdigitated bilayer‐type SmA structures (Figure 16).

### 3.3 Effect of Fluorine Substitution Pattern on Mesophase Stability

A systematic comparison of Series **4** and **5** revealed that the fluorine substitution pattern on the mesogenic core significantly influenced the mesophase stability. In particular, the *T*_m_ and *T*_c_ values of the 3,5‐difluorinated compounds (Series **5**) were consistently higher than those of their corresponding 2,3‐difluorinated analogs (Series **4**). This trend suggests that fluorination at the 3,5‐positions stabilizes both the Cr and SmA phases more effectively than fluorination at the 2,3‐positions, likely owing to differences in their molecular symmetry, linearity, and packing efficiency. These results demonstrate that not only the number of fluorine atoms but also their precise positions within the mesogenic core play a decisive role in governing mesomorphic behavior.

### 3.4 Aggregation Modes Governed by Fluorine Content

In addition to the fluorination of the mesogenic core, the fluorine content of the semifluoroalkoxy flexible chains exerted a pronounced effect on molecular aggregation and phase behavior. The **8F4H** series, of which the flexible chains had a higher fluorine ratio, exhibited markedly higher *T*_m_ and *T*_c_ values than the corresponding **4F6H** series. This behavior can be rationalized in terms of the distinct aggregation modes of the fluoroalkyl segments. Fluoroalkyl chains shorter than approximately (CF_2_)_7_ are dominated primarily by dipole–dipole interactions [79], whereas longer fluoroalkylene segments are known to form two‐dimensional network structures through interchain aggregation, as described by the Stratified Dipole Arrays (SDA) theory [80, 81]. In the present system, the increased fluorine content of the flexible chains promoted chain–chain aggregation, which enhanced the layered ordering but simultaneously altered the balance of intermolecular interactions within the mesogenic cores.

### 3.5 Photoluminescence across the Iso, SmA, and Cr Phases

**FIGURE 17:**
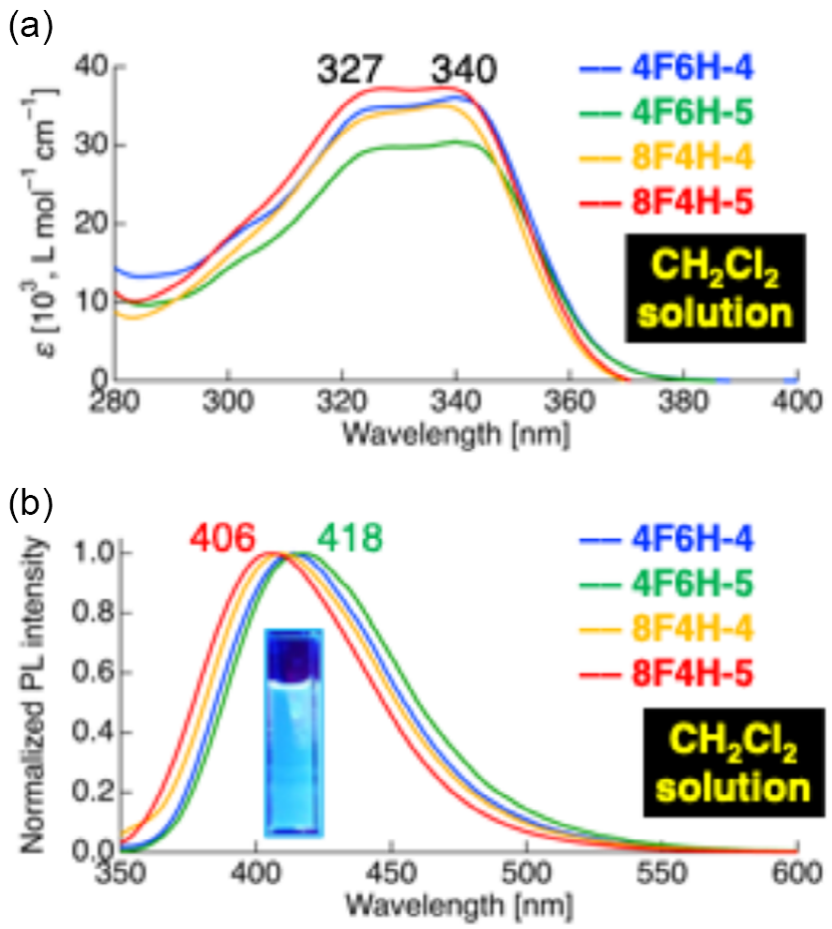
(a) UV–vis absorption and (b) photoluminescence (PL) spectra of **4F6H‐4**, **4F6H‐5**, **8F4H‐4**, and **8F4H‐5** in CH_2_Cl_2_ solution, shown as representative examples. Concentrations: 1.0 × 10^−5^ mol L^−1^ for absorption and 1.0 × 10^−6^ mol L^−1^ for PL measurements.

The photophysical properties of difluorinated tolane derivatives **4** and **5** were evaluated in dilute CH_2_Cl_2_ solution, the Cr state, and during the phase transition (Table 2). In CH_2_Cl_2_ solution, all compounds exhibited two absorption maxima at *λ*_abs_ ≈326–329 nm and 337–340 nm, regardless of the fluorine substitution pattern on the mesogenic core or the fluorine content of the flexible chains (Figure 17a). TD‐DFT calculations revealed that these absorption bands originate from π–π* transitions from the HOMO to the LUMO.

**TABLE 2:** Photophysical properties of 4F6H‐4, 4F6H‐5, 8F4H‐4, and 8F4H‐5 in CH_2_Cl_2_ solution and in the crystalline state.

	Solution			Crystal	
Compound	*λ*_abs_ [nm] (*ε* [10^3^, L mol^–1^ cm^–1^]) a	*λ*_PL_ [nm] b	Φ_PL_ c	*λ*_PL_ [nm]	Φ_PL_ c
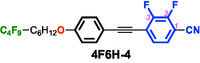	327 (34.8), 340 (36.1)	414	0.18	383*sh*, 401, 424, 445*sh*	0.65
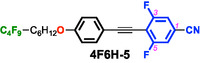	329 (29.8), 340 (30.5)	418	0.26	383*sh*, 399, 424, 445*sh*	0.60
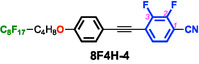	326 (33.9), 337 (35.0)	410	0.20	386, 401*sh*, 435*sh*	0.15
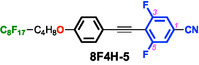	327 (37.2), 338 (37.5)	406	0.28	381, 398*sh*, 424*sh*	0.27

a Concentration: 1.0 × 10^–5^ mol L^–1^;

b Concentration: 1.0 × 10^–6^ mol L^–1^;

c Determined using an absolute photoluminescence quantum yield measurement system with an integrating sphere. *sh.* = shoulder peak.

Upon photoexcitation, all compounds emitted blue fluorescence with emission maxima at *λ*_PL_ = 406–418 nm and their Φ_PL_ values of 0.18–0.28. Although the positions of the fluorine substituents on the mesogenic core had little influence on the emission wavelengths, increasing the fluorine content of the flexible chains led to systematic blueshifts, reflecting the stronger electron‐withdrawing nature of the highly fluorinated alkyl groups.

**FIGURE 18:**
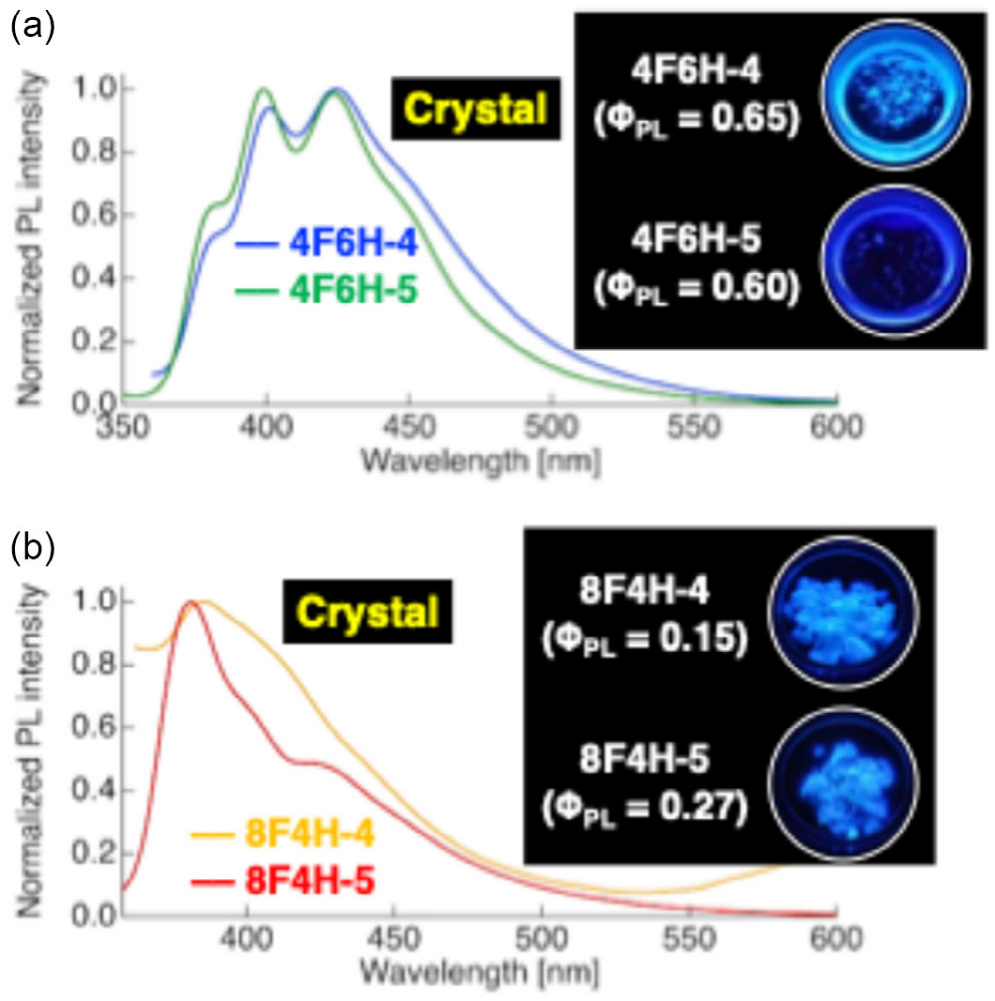
PL spectra and corresponding photographs of solid‐state photoluminescence under UV irradiation for (a) **4F6H‐4** (*λ*_ex_ = 340 nm), **4F6H‐5** (*λ*_ex_ = 340 nm), and (b) **8F4H‐4** (*λ*_ex_ = 320 nm), **8F4H‐5** (*λ*_ex_ = 348 nm) in the Cr state.

In the Cr state, the PL behavior strongly depended on the fluorine content of the flexible chains (Figure 18).

**FIGURE 19:**
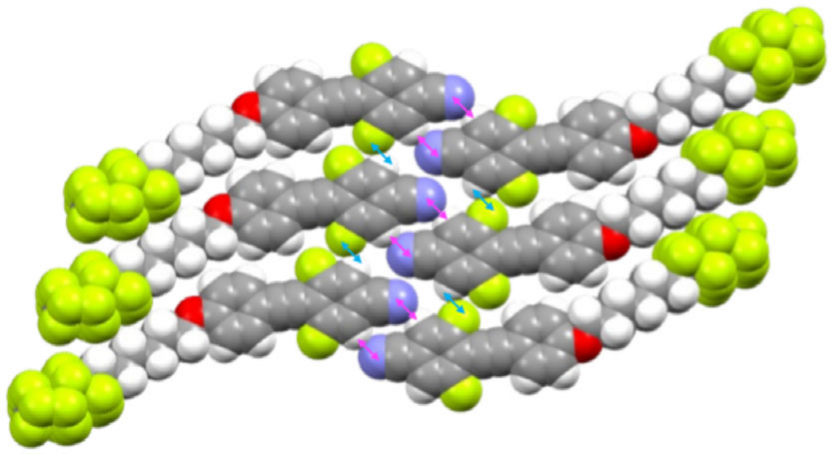
Crystal packing structures of **4F6H‐5**. ↔: short N···H contact (246.6 pm); ↔: short C–H···F contact (250.2 pm). Gray: C; green: F; blue: N; red: oxygen; white: hydrogen.

The **4F6H** series exhibited higher FPL values (0.60–0.65) than the **8F4H** series (0.15–0.27), indicating that excessive chain aggregation in the latter weakens intermolecular interactions between mesogenic cores. Single‐crystal XRD analysis of **4F6H‐5** (Figure 19; CCDC 22 937) revealed the presence of C–H···F (short contact: 250.2 pm) and N···H intermolecular interactions (short contact: 246.6 pm) between mesogenic cores, which effectively suppress molecular motion and enhance Φ_PL_.

**FIGURE 20:**
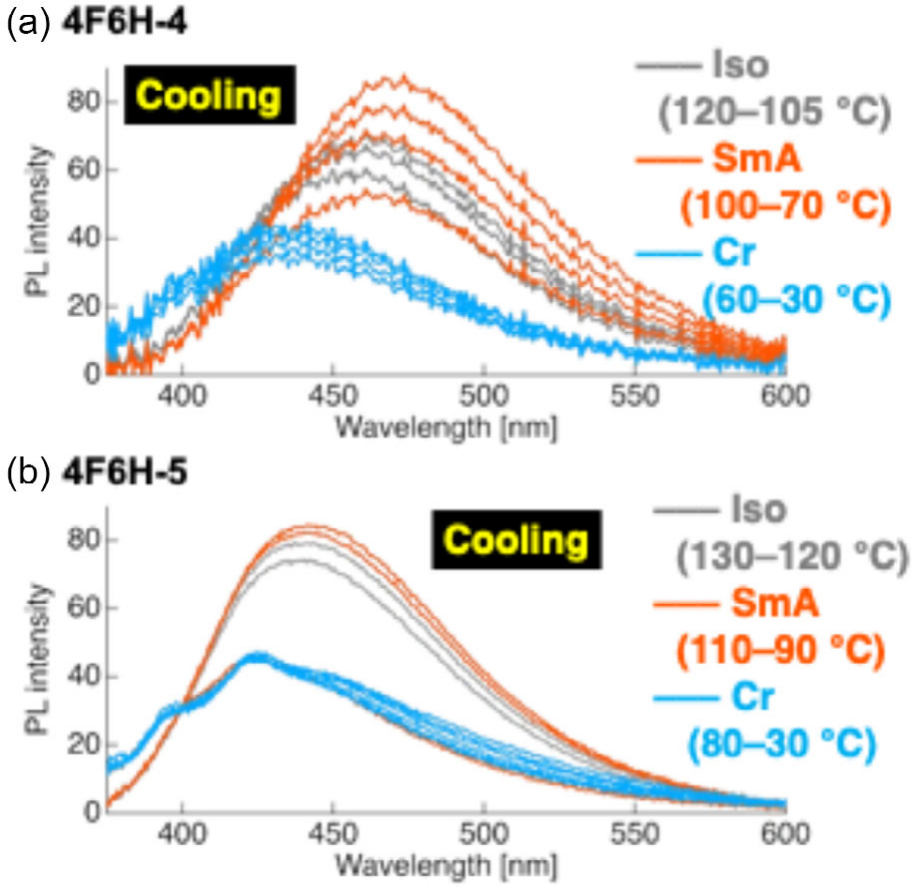
Evolution of PL behavior of compounds (a) **4F6H‐4** and (b) **4F6H‐5** during the Iso → SmA → Cr phase transitions upon cooling.

The temperature‐dependent PL behavior during cooling from the Iso phase further highlights the dynamic interplay between the phase transitions and emission (Figure 20).

For both **4F6H‐4** and **4F6H‐5**, partial recovery of Φ_PL_ was observed at the Iso → SmA transition, whereas crystallization led to a marked decrease in Φ_PL_. This reflects the differences in the packing structure formed during recrystallization and cooling‐induced crystallization.

### 3.6 Design Insights from Partially Fluorinated Tolane Systems

The results presented in this section demonstrate that partial fluorination of tolane‐based mesogens provides a powerful means of balancing mesophase formation and photoluminescence. An optimal fluorination level allows the formation of stable SmA phases while preserving strong solid‐state emission, whereas excessive fluorination of flexible chains promotes chain–chain aggregation that suppresses Φ_PL_. Importantly, both the fluorine substitution pattern on the mesogenic core and the fluorine content of the flexible chains must be considered in concert to control the molecular aggregation hierarchies. These findings highlight partial fluorination as a versatile and complementary design strategy for molecular dimerization to construct compact photoluminescent liquid crystals with finely tunable structure–property relationships.

## 4 Ionic Functionalization as a Strategy for Dynamic Layered Order and Photoluminescence Control

In Section 3, the partial fluorination of tolane‐based mesogens combined with flexible semifluoroalkoxy chains was shown to stabilize the SmA phases through enhanced chain–chain aggregation [72]. While this approach effectively promotes layered ordering, it also has an inherent limitation: excessive aggregation of flexible chains can weaken the intermolecular interactions between mesogenic cores, thereby reducing the PL efficiency. These observations motivated us to explore a third complementary design strategy based on ionic functionalization, in which cohesive electrostatic interactions are introduced at the termini of flexible chains to stabilize layered mesophases while preserving dynamic molecular mobility.

### 4.1 Design Concept: Ionic Cohesion to Stabilize Layered Mesophases

The introduction of an ionic group at the terminal end of a flexible chain is an effective strategy for enhancing cohesive interactions without relying solely on fluorine‐driven aggregation [82, 83, 84]. In contrast to semifluoroalkoxy chains, which stabilize layered structures primarily through dipolar and van der Waals interactions, an ionic group is expected to generate strong electrostatic cohesion and promote the formation of robust ionic sublayers within the Sm phases. Such ionic cohesion was anticipated to stabilize the SmA phases over a broad temperature range to potentially extend the mesophase stability to near room temperature. Moreover, because the ionic group is spatially separated from the π‐conjugated mesogenic core, this approach offers the possibility of independently tuning the mesomorphic order and photophysical properties. Based on these considerations, we focused on incorporating an imidazolium salt terminus into the difluorinated tolane‐based mesogens.

### 4.2 Synthetic Considerations and Design Constraints

**SCHEME 1:**
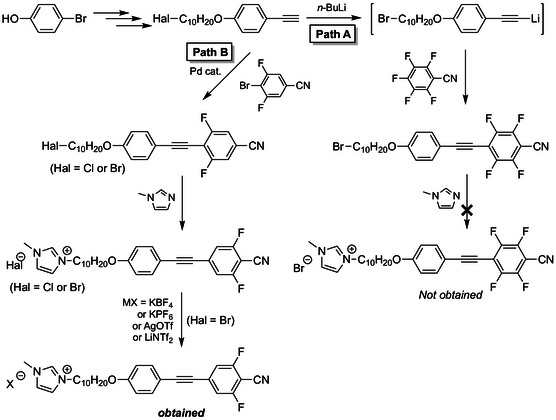
Synthetic routes to a tetrafluorinated tolane bearing a flexible chain with a terminal imidazolium salt (**Path A**) and to its difluorinated analog (**Path B**).

Our initial synthetic efforts were directed toward the preparation of tetrafluorinated tolane derivatives bearing a flexible chain with a terminal imidazolium salt, starting from highly emissive tetrafluorinated tolane **10H‐A** (Scheme 1, Path A).

However, the introduction of an imidazolium group via an S_*N*_2 reaction between this precursor and *N*‐methylimidazole under basic conditions was unsuccessful. ^19^F NMR analysis of the reaction mixture indicated that the nucleophilic addition–elimination of *N*‐methylimidazole preferentially occurred at the 2‐ and 5‐positions of the fluorinated aromatic ring, rather than at the terminal bromide. This outcome highlighted a critical design constraint: fluorine substituents at the 2‐ and 5‐positions of the tolane framework significantly increased the susceptibility to nucleophilic aromatic substitution, thereby hindering the introduction of ionic termini. Consequently, the synthetic strategy was revised to include difluorinated tolane mesogens without fluorine substituents at the 2‐ and 5‐positions (Scheme 1, Path B) [73].

**FIGURE 21:**
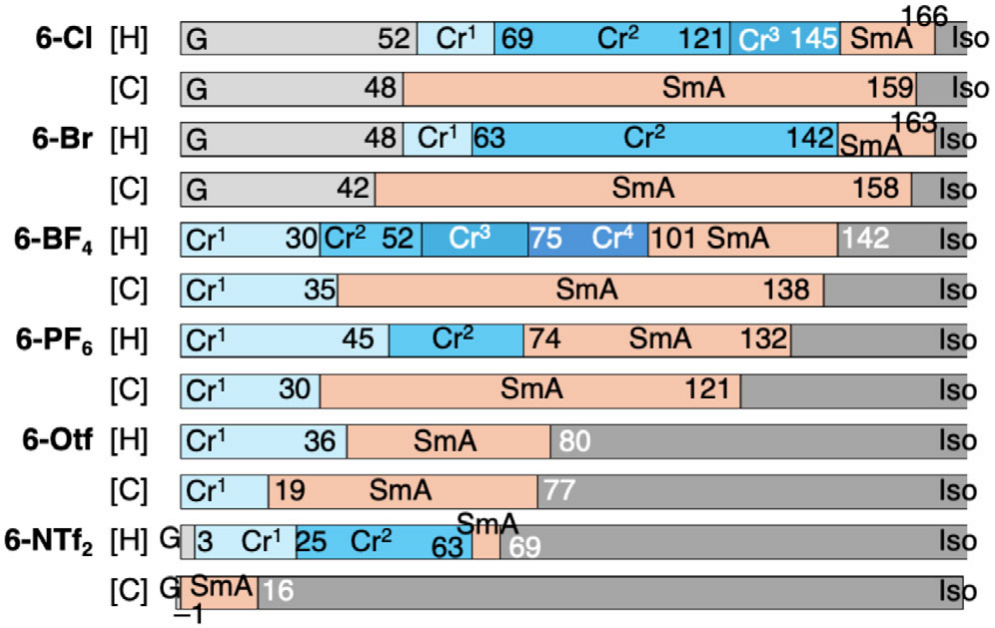
Phase‐transition behaviors of **6‐Cl**, **6‐Br**, **6‐BF**_**4**_, **6‐PF**_**6**_, **6‐OTf**, and **6‐NTf**_**2**_ based on DSC results recorded under N_2_ at a scan rate of 5.0°C min^−1^.

Using this approach, 4‐(10‐halodecyloxy)phenylacetylene (bearing Cl or Br at the terminal position) was coupled with 4‐bromo‐3,5‐difluorobenzonitrile via Pd‐catalyzed Sonogashira coupling to afford the difluorinated tolane precursors. Subsequent S_*N*_2 substitution with *N*‐methylimidazole proceeded smoothly to yield the corresponding imidazolium bromide and chloride salts (6‐Br and 6‐Cl). Anion‐exchange reactions of **6‐Br** with various metal salts, such as KBF_4_ **,** KPF_6_ **,** AgOTf**,** and LiNTf_2_, afforded a series of ionic compounds (**6‐X**; X = BF_4_, PF_6_, OTf, and NTf_2_), which were purified by reprecipitation and recrystallization and subjected to detailed structural, thermal, and photophysical analyses (Figure 21).

### 4.3 Counter‐Anion‐Dependent Mesophase Stability

**FIGURE 22:**
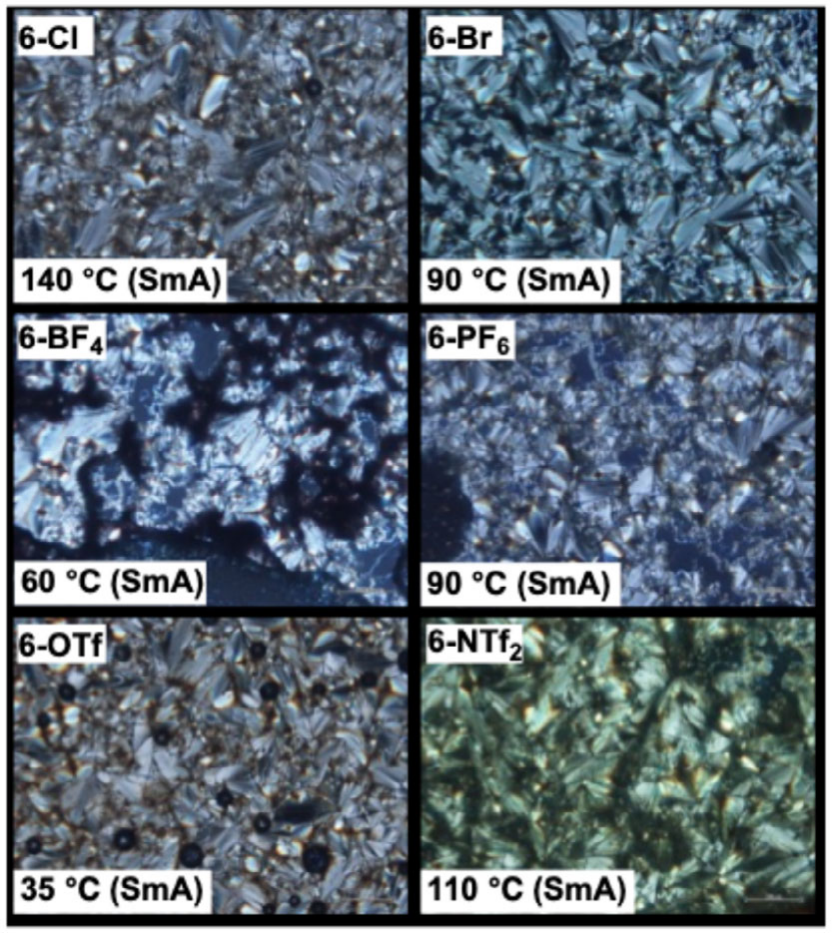
POM textures observed in the mesophase of **6‐Cl**, **6‐Br**, **6‐BF**_**4**_, **6‐PF**_**6**_, **6‐OTf**, and **6‐NTf**_**2**_.

DSC and POM analyses revealed that all **6‐X** compounds exhibited enantiotropic LC behavior, forming mesophases during both the heating and cooling processes, irrespective of the counteranionic species (Figures 21 and 22).

**FIGURE 23:**
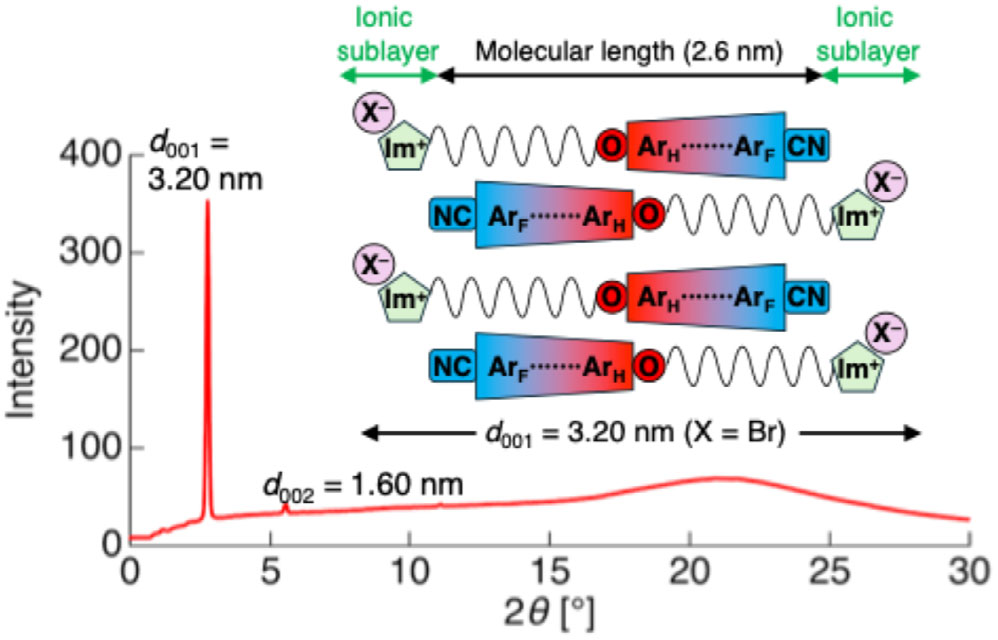
PXRD pattern of **6‐Br** (as a representative example) acquired at 100°C during the cooling process, together with the proposed molecular aggregation structure in the SmA phase. The colors in the figure represent the electron‐density distribution, where red indicates an electron‐rich region and blue indicates electron‐deficient regions.

POM observations showed characteristic fan‐shaped textures that transformed into dark fields upon the application of shear stress. This was indicative of SmA phases (Figure 22), the formation of which was further confirmed by VT‐PXRD measurements (Figure 23).

A representative example (**6‐Br**) had X‐ray diffraction peaks corresponding to the (001) and (002) reflections, yielding a layer spacing (*d*_001_) of 3.20 nm. Considering the calculated molecular length of ≈2.6 nm and the additional thickness associated with the ionic sublayers (≈0.6 nm), the mesophase was identified as an interdigitated bilayer‐type SmA structure.

**FIGURE 24:**
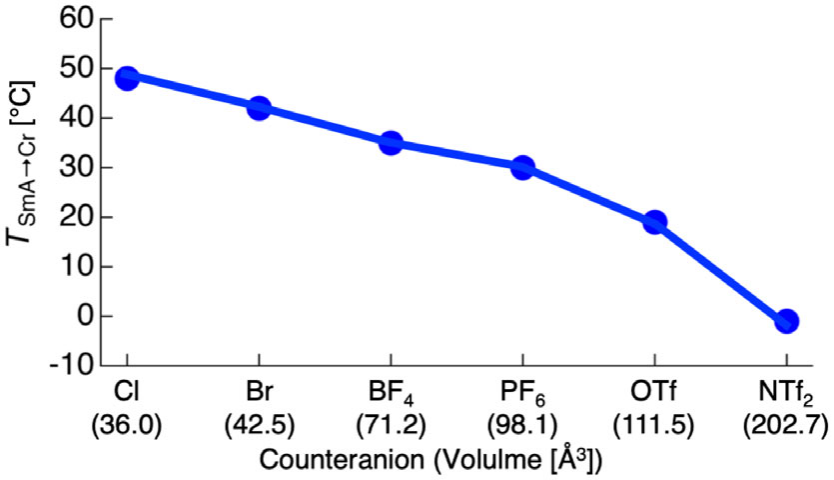
Correlation between the size of the counter anion (X) and the temperature of the phase transition *T*_SmA→Cr_ from the SmA phase to glassy or crystalline phases upon cooling.

A notable feature of these ionic systems was the strong dependence of the SmA‐to‐Cr phase transition temperature (*T*_SmA→Cr_) on the size of the counteranion (Figure 24).

For relatively small anions, such as Cl^‐^ and Br^‐^, *T*_SmA→Cr_ values of 48°C and 42°C, respectively, were observed. As the size of the anion increased, *T*_SmA→Cr_ decreased progressively to 35°C (BF_4_^‐^)**,** 30°C (PF_6_^‐^)**,** 19°C (OTf^‐^), and –1°C (NTf_2_^‐^) [85]. These results demonstrate that selection of the counter anion is a powerful tool for stabilizing the SmA phase over a wide temperature range, including near room temperature.

### 4.4 Anion Effects on Aggregation and Solid‐State Photoluminescence

**FIGURE 25:**
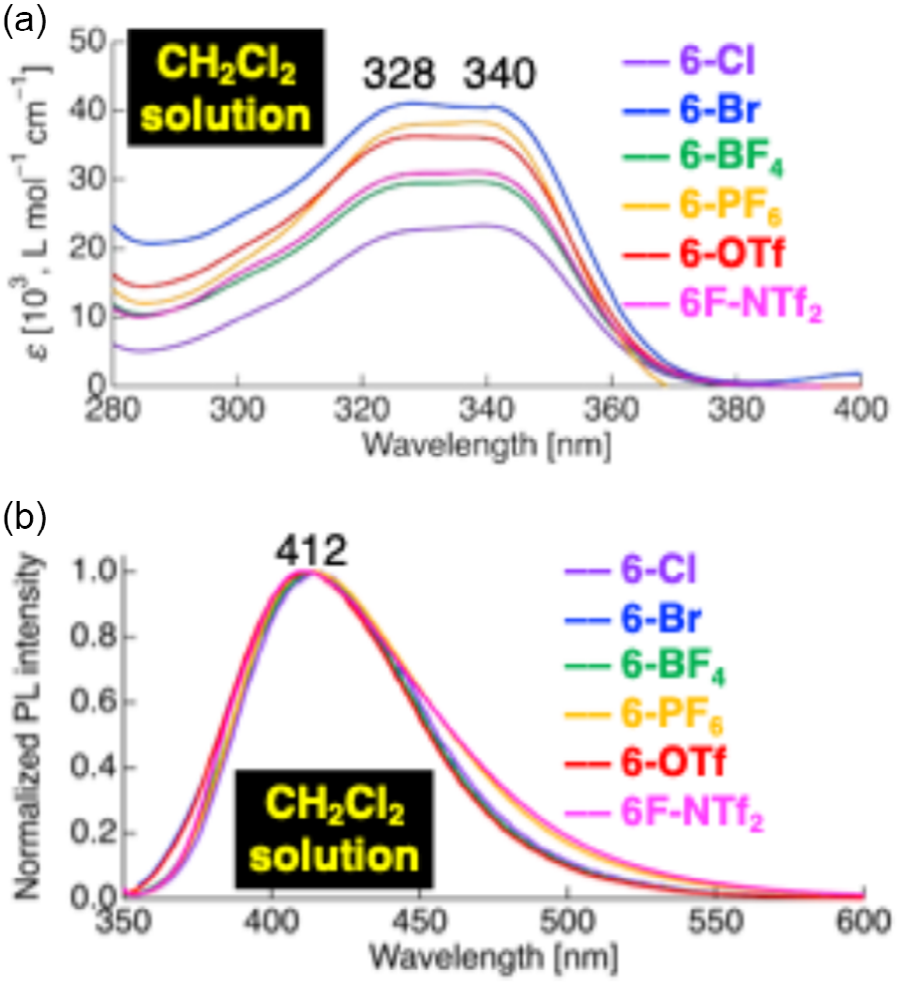
(a) UV–vis absorption and (b) PL spectra of **6‐Cl**, **6‐Br**, **6‐BF**_**4**_, **6‐PF**_**6**_, **6‐OTf**, and **6‐NTf**_**2**_ in CH_2_Cl_2_ solution, shown as representative examples. Concentrations: 1.0 × 10^−5^ mol L^−1^ for absorption and 1.0 × 10^−6^ mol L^−1^ for PL measurements.

The photophysical properties of the ionic compound **6‐X** were examined to elucidate the role of counteranions in modulating molecular aggregation and solid‐state PL behavior. In dilute CH_2_Cl_2_ solution, all compounds exhibited two distinct absorption bands at *λ*_abs_ ≈ 328 nm and 337–340 nm, with negligible dependence on the counteranion (Figure 25a). TD‐DFT calculations indicated that these absorptions arise from π–π* transitions localized on the difluorinated tolane‐based mesogenic core, consistent with the observed anion‐independent solution‐phase absorption behavior.

Upon photoexcitation, all compounds emitted blue fluorescence with PL maxima *λ*_PL_ at 410–415 nm and Φ_PL_ values of 0.21–0.24 (Figure 25b). These Φ_PL_ values are comparable to those of the nonionic difluorinated tolane derivatives 5 series viz. **4F6H‐5** and **8F4H‐5**, as discussed in Section 3, confirming that the intrinsic emissive properties originate from intramolecular charge transfer (ICT) transitions associated with the difluorinated tolane‐based π‐conjugated mesogenic core (Table 3).

**TABLE 3:** Photophysical properties of the 6‐X series in CH_2_Cl_2_ solution and in the crystalline state.

	Solution			Crystal	
Compound	*λ*_abs_ [nm] (*ε* [10^3^, L mol^–1^ cm^–1^]) a	*λ*_PL_ [nm] b	Φ_PL_ c	*λ*_PL_ [nm]	Φ_PL_ c
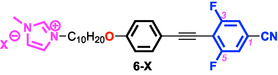					
**6‐Cl**	328 (22.6), 340 (23.4)	414	0.21	402	0.05
**6‐Br**	328 (41.1), 340 (40.4)	412	0.24	371*sh*, 393	0.07
**6‐BF**_**4**_	328 (29.4), 338 (29.7)	415	0.21	402	0.05
**6‐PF**_**6**_	328 (38.0), 338 (38.4)	413	0.24	413	0.42
**6‐OTf**	328 (36.3), 337 (36.2)	410	0.22	438	0.17
**6‐NTf**_**2**_	328 (30.9), 338 (31.1)	412	0.24	465	0.28

a Concentration: 1.0 × 10^–5^ mol L^–1^;

b Concentration: 1.0 × 10^–6^ mol L^–1^;

c Determined using an absolute photoluminescence quantum yield measurement system with an integrating sphere. *sh.* = shoulder peak.

**FIGURE 26:**
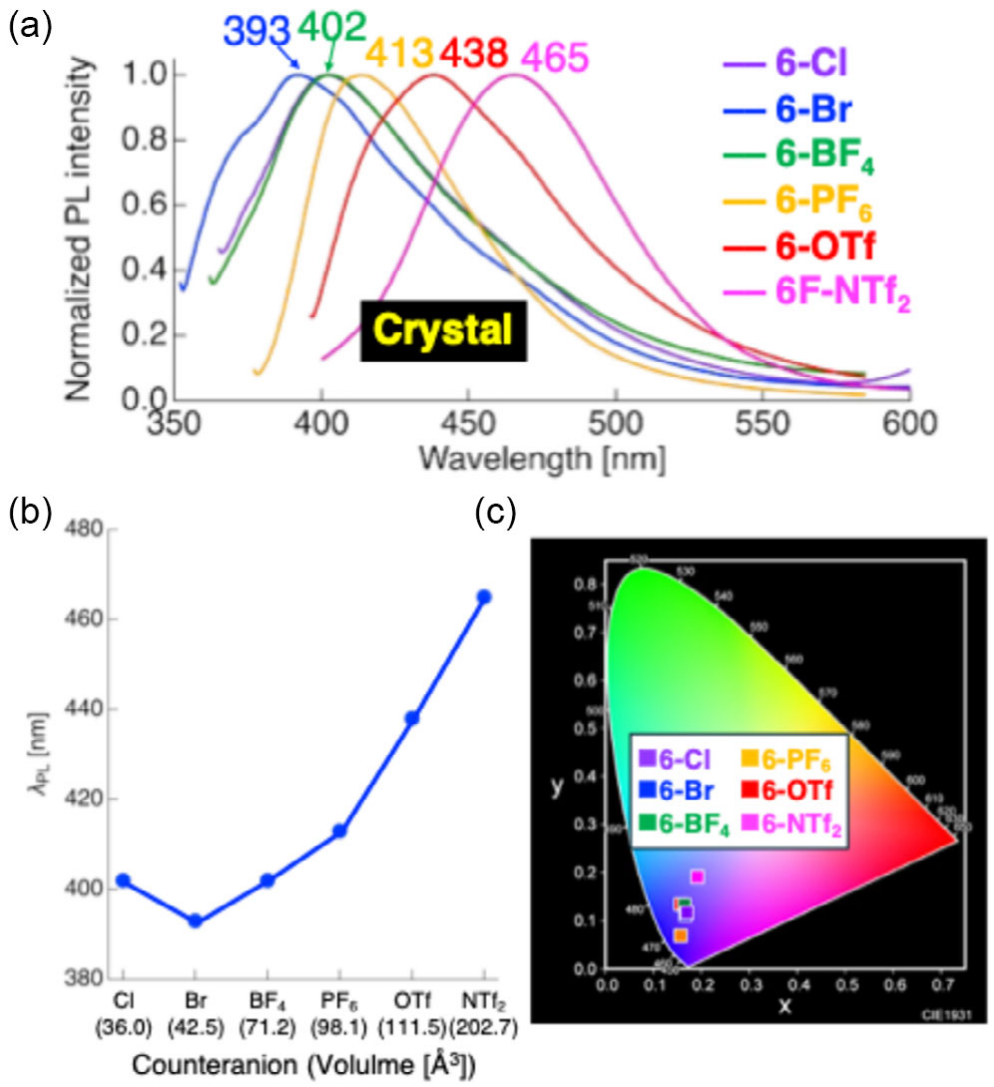
(a) PL spectra of **6‐Cl** (*λ*_ex_ = 331 nm), **6‐Br** (*λ*_ex_ = 331 nm), **6‐BF**_**4**_ (*λ*_ex_ = 331 nm), **6‐PF**_**6**_ (*λ*_ex_ = 358 nm), **6‐OTf** (*λ*_ex_ = 376 nm), and **6‐NTf**_**2**_ (*λ*_ex_ = 376 nm) in the crystalline state. (b) Correlation between the size of counter anions (X) and λ_PL_ for the **6‐X** series. (c) CIE chromaticity diagram showing the PL colors derived from the corresponding PL spectra.

In contrast, the PL behavior of ionic compounds **6‐X** in the Cr state pronouncedly depended on the counteranion species (Figure 26). Both *λ*_PL_ and the spectral profile varied systematically with the anion size, with *λ*_PL_ spanning a wide range from 393–465 nm (Figure 26a). In general, bulkier anions induced progressive redshift in the emission (Figure 26b), resulting in diverse solid‐state emission colors ranging from deep blue to bright blue (Figure 26c).

Furthermore, the Φ_PL_ values were significantly enhanced for the compounds bearing bulky anions (PF_6_^–^, OTf^–^, and NTf_2_^–^; Φ_PL_ = 0.17–0.42) compared with those containing smaller anions (Cl^–^, Br^–^, BF_4_^–^; Φ_PL_ = 0.05–0.07). This enhancement is attributed to the tighter packing of the π‐conjugated mesogenic cores facilitated by bulky anions, which suppress molecular motion and reduce nonradiative deactivation pathways.

### 4.5 Photoluminescence across Iso, SmA, and Cr Phases

**FIGURE 27:**
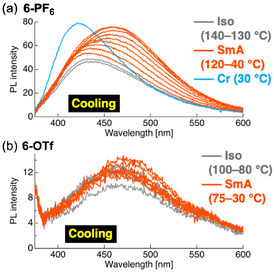
Evolution of PL behavior of compounds (a) **6‐PF**_**6**_ and (b) **6‐OTf** during the Iso → SmA → Cr phase transitions upon cooling.

The temperature‐dependent PL behavior of the representative ionic compounds **6‐PF**_**6**_ and **6‐OTf** was investigated by cooling these compounds from the Iso phase through the SmA and Cr phases (Figure 27).

In both systems, Φ_PL_ was observed to partially recover at the Iso → SmA transition, which reflected the formation of ordered layered structures that restrict molecular motion.

For compound **6‐PF**_**6**_, the Iso phase at 140°C had an emission maximum at *λ*_PL_ ≈ 422 nm with Φ_PL_ = 0.11. Upon cooling to the SmA phase (120°C), Φ_PL_ increased slightly, followed by a further increase upon cooling to lower temperatures within the SmA phase. Subsequent crystallization caused Φ_PL_ to decrease and *λ*_PL_ was blue‐shifted, thus approaching the emission characteristics of recrystallized samples. A similar trend was observed for **6‐OTf**, although the absolute Φ_PL_ values were lower. These results indicate that the PL behavior of ionic tolane systems is highly sensitive to the phase‐formation pathway, with distinct aggregation structures formed during recrystallization and cooling‐induced crystallization.

### 4.6 Design Insights from Ionic Tolane‐Based PLLCs

The results presented in this section establish ionic functionalization as a powerful strategy for stabilizing layered mesophases and modulating the solid‐state photoluminescence in tolane‐based photoluminescent liquid crystals. The introduction of imidazolium salt termini enables the formation of robust SmA phases irrespective of the counteranion, while precise tuning of the mesophase stability to near‐room temperature can be achieved through rational counteranion selection. Importantly, bulky counteranions not only stabilize smectic ordering but also enhance the PL efficiency by promoting the favorable aggregation of the π‐conjugated mesogenic cores. These findings demonstrate that ionic interactions provide a distinct design parameter complementary to molecular dimerization and partial fluorination, and enables hierarchical control over the molecular order and photophysical properties of compact PLLC systems.

## 5 Conclusion and Outlook

### 5.1 Conceptual Summary: Three Complementary Design Strategies

In this account, we demonstrated that the long‐standing trade‐off between mesomorphism and solid‐state PL in compact π‐conjugated systems can be effectively mitigated by using three complementary molecular design strategies based on fluorinated tolane frameworks.

First, molecular dimerization was shown to be an effective means of quelling the crystallinity while preserving the intrinsic emissive properties of fluorinated tolane‐based mesogens. By introducing flexible spacers between two identical mesogenic units, crystallization could be suppressed and LC phases could be induced, thus enabling the dynamic modulation of PL across crystalline and mesomorphic states.

Second, partial fluorination of the mesogenic core combined with the introduction of semifluoroalkoxy flexible chains provides a powerful strategy for balancing the mesophase stability and emission efficiency. These studies revealed that not only the number of fluorine atoms but also their precise positions on the tolane framework and their distribution between the core and flexible chains critically govern molecular aggregation, and hence the mesophase and PL behavior.

Third, ionic functionalization using imidazolium‐salt‐terminated flexible chains introduced electrostatic cohesion as an alternative ordering force. This strategy enabled the robust stabilization of SmA phases over a wide temperature range, including near room temperature, while counter‐anion selection offers an additional means for tuning both the mesophase stability and solid‐state PL emission.

Even though each of these strategies addresses molecular order and emission from a different perspective, all three converge toward the same objective: the realization of simultaneous stable mesomorphic order and controllable PL within compact molecular architectures.

### 5.2 General Design Guidelines for Photoluminescent Liquid Crystals

Several general design guidelines for PLLCs can be derived from the systematic investigations described in this account.

(i) Electronic and geometric control via fluorination.

The position and number of fluorine substituents exert a dual influence on the electronic density distribution and molecular geometry. Appropriate fluorination can enhance the PL while maintaining sufficient molecular linearity to support mesophase formation.

(ii) Decoupling crystallinity and mesomorphism with flexible elements.

The spacer length, terminal chain structure, and introduction of ionic termini function as adjustable parameters to subdue excessive crystallinity without disrupting the emissive‐conjugated mesogenic core.

(iii) Hierarchical control of molecular aggregate formation

The balance between intermolecular mesogenic core−core interactions and the aggregation of flexible chains or ionic sublayers plays a decisive role in determining both LC and PL behaviors. Rational molecular design must therefore consider these competing aggregation modes simultaneously.

Together, these guidelines establish a versatile framework for engineering PLLCs with finely tunable structure–property relationships.

### 5.3 Implications and Scope beyond Tolane Systems

These studies focused on fluorinated tolane‐based systems, but the design principles elucidated herein are not limited to this specific framework. Tolane serves as a particularly instructive model owing to its compact, rigid, and electronically well‐defined π‐conjugated structure; however, the same concepts—dimerization, balanced fluorination, and ionic functionalization—are expected to be broadly applicable to other π‐conjugated mesogenic systems, including stilbene derivatives, phenyl–ethynyl heteroaromatic frameworks, and ionic liquid–crystalline luminophores. Accordingly, the strategies described in this account provide a general conceptual platform for the rational design of compact photoluminescent soft materials that integrate molecular ordering with optical functionality.

### 5.4 Future Directions and Outlook

The sharp phase transitions associated with the narrow mesophase windows of some of the compounds presented here are advantageous for temperature‐triggered photoluminescence switching and sensing applications, rather than for broad‐range display devices. From this perspective, the present systems are more appropriately suited as stimuli‐responsive photonic materials than conventional display technologies. Despite the progress described herein, several challenges remain to be resolved before PLLCs could be advanced from empirical design to predictive molecular engineering. In particular, the PL efficiency in LC phases often remains lower than that in the Cr state, which reflects the complex interplay between molecular mobility, aggregation, and excited‐state relaxation. Future efforts aimed at elucidating the intrinsic relationship between aggregated molecular structures and photophysical processes through time‐resolved spectroscopic techniques, in situ structural analysis, and molecular dynamics simulations are essential for a deeper understanding. These studies are expected to clarify the extent to which dynamic molecular ordering in LC phases influences nonradiative deactivation pathways and emission efficiency. From an application perspective, fluorinated tolane‐based PLLCs developed using these design strategies show promise as temperature‐responsive light‐emitting materials, stimulus‐responsive photonic systems, ion‐conductive photofunctional materials, and related soft electronic devices. The continued integration of molecular design, structural characterization, and photophysical analysis is foreseen to further expand the functional scope of PLLCs.

## Supporting Information

Additional supporting information can be found online in the Supporting Information section.

## Author Contributions

S.Y. conceptualized and designed the study, performed the data analysis, and wrote the manuscript. M.Y. and T.K. contributed to manuscript preparation, critical review, and editing. All the authors discussed the results and approved the final manuscript.

## Conflicts of Interest

The authors declare no conflicts of interest.

## Supporting information

Supplementary Material
