# Supplementary material for: Design Strategies for Compact Photoluminescent Liquid Crystals Based on Fluorinated Tolane Frameworks
Source: Chem Rec. 2026 Mar 31;26(5):e202500346. doi: 10.1002/tcr.202500346 (PMC13184814; doi:10.1002/tcr.202500346)
Supplement: Supplementary file 1 — Supplementary Material [file TCR-26-e202500346-s001.pdf]

Supporting Information

Design Strategies for Compact Photoluminescent Liquid Crystals Based on Fluorinated Tolane Frameworks

Shigeyuki Yamada, Motohiro Yasui, Tsutomu Konno*

Matsugasaki, Sakyo-ku, Kyoto 606-8585, Japan
E-mail: syamada@kit.ac.jp

Compounds' Characterization Data

1,6-Bis[4-[2-(2,3,5,6-tetrafluoro-4-hexyloxyphenyl)ethyn-1-yl]phenoxy]hexane (C6-1*6*)[1]

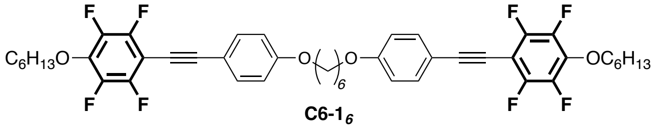

Yield: 59%; T_c: 160 °C (on the 1st cooling process); ^{1}H NMR (CDCl$_3$): δ 0.91 (t, J = 6.8 Hz, 6H), 1.26–1.40 (m, 8H), 1.47 (quint., J = 6.8 Hz, 4H), 1.52–1.60 (m, 4H), 1.73–1.92 (m, 8H), 4.00 (t, J = 6.4 Hz, 4H), 4.25 (t, J = 6.4 Hz, 4H), 6.88 (d, J = 8.8 Hz, 4H), 7.49 (d, J = 8.8 Hz, 4H); ^{13}C NMR (CDCl$_3$): δ 14.0, 22.5, 25.2, 25.8, 29.1, 29.8, 31.4, 67.9, 72.9, 75.5 (t, J = 3.6 Hz), 98.4 (t, J = 12.4 Hz), 100.5 (t, J = 3.6 Hz), 113.9, 114.6, 133.4, 137.8 (t, J = 12.4 Hz), 141.1 (ddt, J = 250.2, 10.3, 5.8 Hz), 147.1 (dm, J = 257.5 Hz), 159.9; ^{19}F NMR (CDCl$_3$, BTF): δ –138.78 (dd, J = 21.8, 6.8 Hz, 4F), –157.87 (dd, J = 21.8, 6.8 Hz, 4F); IR (KBr): ν 2979, 2883, 2273, 1737, 1524, 1460, 1265, 1179, 1108, 927, 854 cm^{-1};HRMS (FAB) calcd for [M$^+$] $C_{46}H_{46}F_8O_4$: 814.3268, found: 814.3260.

1,8-Bis[4-[2-(2,3,5,6-tetrafluoro-4-hexyloxyphenyl)ethyn-1-yl]phenoxy]octane (C6-1*8*)[1]

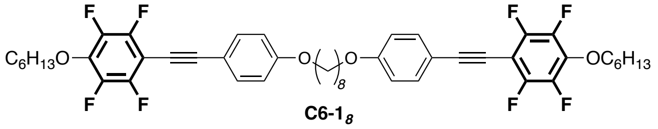

Yield: 41%; T_m: 129 °C, T_c: 132 °C (on the 1st cooling process); ^{1}H NMR (CDCl$_3$): δ0.91 (t, J = 6.8 Hz, 6H), 1.28–1.54 (m, 20H), 1.79 (dq, J = 15.2, 8.4 Hz, 8H), 3.98 (t, J = 6.4 Hz, 4H), 4.25 (t, J = 6.4 Hz, 4H), 6.88 (d, J = 8.8 Hz, 4H), 7.49 (d, J = 8.8 Hz, 4H); ^{13}C NMR (CDCl$_3$): δ 14.0, 22.5, 25.2, 26.0, 29.1, 29.3, 29.8, 31.4, 68.1, 72.9 (t, J = 3.7 Hz), 75.5 (t, J = 3.6 Hz), 98.4 (t, J = 17.5 Hz), 100.5 (t, J = 3.6 Hz), 113.8, 114.6, 133.4, 137.8 (tt, J = 13.2, 2.9 Hz), 139.7–142.6 (dm, J = 246.5 Hz), 145.7–148.6 (dm, J = 248.0 Hz), 159.9; ^{19}F NMR (CDCl$_3$, BTF): δ–138.80 (dd, J = 21.8, 6.8 Hz, 4F), –157.88 (dd, J = 21.8, 6.8 Hz, 4F); IR (KBr): ν 2922, 2858, 2211, 1604, 1490, 1248, 1173, 984, 840 cm^{-1};HRMS (FAB) calcd for [M$^+$] $C_{48}H_{50}F_8O_4$: 842.3581, found: 842.3572.

1,10-Bis[4-[2-(2,3,5,6-tetrafluoro-4-hexyloxyphenyl)ethyn-1-yl]phenoxy]decane (C6-1*10*)[1]

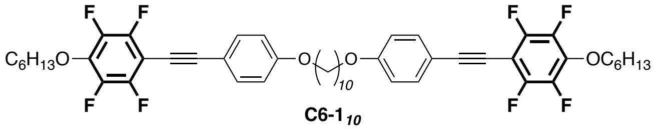

Yield: 43%; T_m: 116 °C, T_c: 124 °C (on the 1st cooling process); ^{1}H NMR (CDCl$_3$): δ0.91 (t, J = 7.2 Hz, 6H), 1.26–1.52 (m, 24H), 1.74–1.85 (m, 8H), 3.98 (t, J = 6.4 Hz, 4H), 4.25 (t, J = 6.4 Hz, 4H), 6.88 (d, J = 8.9 Hz, 2H), 7.49 (d, J = 8.9 Hz, 2H); ^{13}C NMR (CDCl$_3$): δ 14.0, 22.5, 25.2, 26.0, 29.1, 29.3, 29.4, 29.9, 31.4, 68.1, 72.9 (t, J = 3.6 Hz), 75.5, 98.2–98.6 (m), 100.5, 113.8, 114.6, 133.3, 137.8 (t, J = 16.8 Hz), 139.8–142.5 (dm, J = 250.1 Hz), 145.7–148.5 (dm, J = 250.1 Hz), 160.0; ^{19}F NMR (CDCl$_3$, C_6F_6): δ–139.80 (dd, J = 21.8, 6.8 Hz, 4F), –158.89 (dd, J = 21.8, 6.8 Hz, 4F); IR (KBr): ν2936, 2854, 2216, 1604, 1519, 1491, 1292, 1249, 1172, 1018, 984, 830 cm^{-1};HRMS (FAB) calcd for [M$^+$] $C_{50}H_{54}F_8O_4$: 870.3894, found: 870.3891.

[1] S. Yamada, E. Uto, T. Sakurai, T. Konno, *Journal of Molecular Liquids* 362 (2022): 119755..

1,6-Bis(4-[2-(2,3,5,6-tetrafluoro-4-decyloxyphenyl)ethyn-1-yl]phenoxy)hexane (C10-1_{6}) [2]

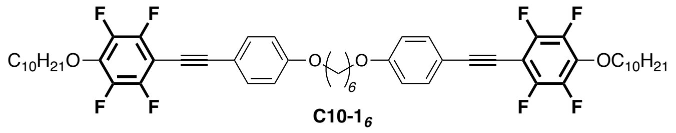

Yield: 16%, white solid, M.p.: 147 °C; ^{1}H NMR (CDCl3): δ 0.88 (t, J = 6.8 Hz, 6H), 1.23–1.40 (m, 26H, C*H2*), 1.46 (quin, J = 7.2 Hz, 4H), 1.72–1.89 (m, 10H), 4.00 (t, J = 6.8 Hz, 4H), 4.25 (t, J = 6.8 Hz, 4H), 6.88 (d, J = 8.8 Hz, 2H), 7.49 (d, J = 8.8 Hz, 4H); ^{13}C NMR ($CDCl_3$): δ 14.1, 22.7, 25.5, 25.8, 29.1, 29.2, 29.3, 29.5 (for two carbons), 29.9, 31.9, 67.9, 72.9 (t, J = 3.7 Hz), 75.5 (t, J = 2.9 Hz), 98.4 (t, J = 17.6 Hz), 100.4 (t, J = 3.0 Hz), 113.9, 114.6, 133.4, 137.8 (t, J = 12.5 Hz), 139.6–142.5 (dm, J = 246.5 Hz), 145.6–148.6 (dm, J = 250.9 Hz), 159.9; ^{19}F NMR(CDCl3, C6F6): δ –139.72 to –139.83 (m, 4F), –158.82 to –158.94 (m, 4F); IR (KBr): ν 2924, 2854, 1606, 1521, 1491, 1252, 1175, 1031 cm^{-1}; HRMS (FAB) Calcd for (M+H) C54H63F4O4: 927.4599, Found: 927.4590.

1,8-Bis(4-[2-(2,3,5,6-tetrafluoro-4-decyloxyphenyl)ethyn-1-yl]phenoxy)octane (C10-1_{8})[2]

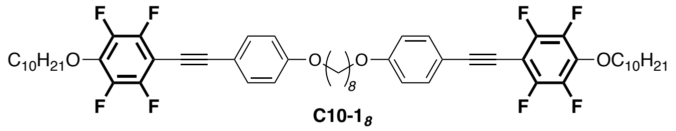

Yield; 65%, white solid; M.p.: 121 °C; ^{1}H NMR (CDCl3): δ 0.88 (t, J = 6.4 Hz, 6H), 1.24–1.37 (m, 26H), 1.40–1.52 (m, 8H), 1.73–1.85 (m, 10H), 3.98 (t, J = 6.0 Hz, 4H), 4.25 (t, J = 6.4 Hz, 4H, OC*H3*), 6.88 (d, J = 8.8 Hz, 4H), 7.49 (d, J = 8.8 Hz, 4H); ^{13}C NMR ($CDCl_3$): δ 14.1, 22.7, 25.5, 25.9, 29.1, 29.2, 29.26, 29.29, 29.5 (for two carbons), 29.9, 31.9, 68.0, 72.9 (t, J = 3.6 Hz), 75.5 (t, J = 2.9 Hz), 98.4 (t, J = 17.6 Hz), 100.5 (t, J = 3.6 Hz), 113.8, 114.6, 133.4, 137.8 (tt, J = 12.5, 2.9 Hz), 139.7–142.5 (dm, J = 247.2 Hz), 145.7–148.6 (dm, J = 250.8 Hz), 159.9; ^{19}F NMR(CDCl3, C6F6): δ –139.72 to –139.84 (m, 4F), –158.82 to –158.93 (m, 4F); IR (KBr): ν 2918, 2854, 1604, 1517, 1491, 1290, 1248, 1172, 1037 cm^{-1}; HRMS (FAB) Calcd for (M+H) C56H67F4O4: 955.4912, Found: 955.4902.

1,10-Bis(4-[2-(2,3,5,6-tetrafluoro-4-decyloxyphenyl)ethyn-1-yl]phenoxy)decane (C10-1_{10})[2]

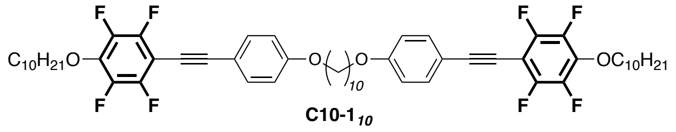

Yield; 5%, white solid; M.p.: 110 °C; ^{1}H NMR (CDCl3): δ 0.88 (t, J = 6.8 Hz, 6H), 1.23–1.40 (m, 30H), 1.45 (quin, J = 7.2 Hz, 8H), 1.72–1.83 (m, 10H), 3.98 (t, J = 6.4 Hz, 4H), 4.24 (t, J = 6.8 Hz, 4H), 6.88 (d, J = 8.8 Hz, 4H), 7.49 (d, J = 8.8 Hz, 4H) ppm; ^{13}C NMR ($CDCl_3$): δ 14.1, 22.7, 25.5, 26.0, 29.1, 29.2, 29.29, 29.33, 29.4, 29.5 (for two carbons), 29.9, 31.9, 68.1, 72.9 (t, J = 3.6 Hz), 75.5 (t, J = 3.6 Hz), 98.4 (t, J = 16.2 Hz), 100.5 (t, J = 3.6 Hz), 113.8, 114.6, 133.3, 137.8 (tt, J = 13.2, 2.9 Hz), 139.6–142.6 (dm, J = 247.2 Hz), 145.7–148.8 (dm, J = 236.1 Hz), 160.0; ^{19}F NMR (CDCl3, C6F6): δ –139.74 to –139.89 (m, 4F), –158.82 to –158.95 (m, 4F); IR (KBr): ν 2922, 2853, 1605, 1517, 1491, 1291, 1248, 1021 cm^{-1}; HRMS (FAB) Calcd for (M^{+}) C58H70F4O4: 982.5146, Found: 982.5151.

---

[2] S. Inui, H. Kitaoka, Y. Eguchi, M. Yasui, T. Konno, S. Yamada, *Crystals* 15 (2025): 840.

1,6-Bis(4-[2-(4-decyloxyphenyl)ethyn-1-yl]2,3,5,6-tetrafluorophenoxy)hexane (2*6*) [3]

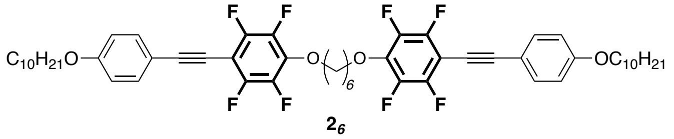

2*6*

Yield; 41%, white solid: M.p.: 98 °C; ^{1}H NMR (CDCl3): *δ* 0.88 (t, *J* = 6.8 Hz, 6H), 1.22–1.40 (m, 26H), 1.46 (quin, *J* = 7.2 Hz, 4H), 1.72−1.88 (m, 10H), 3.98 (t, *J* = 6.4 Hz, 4H), 4.27 (t, *J* = 6.4 Hz, 4H), 6.88 (d, *J* = 8.8 Hz, 4H), 7.49 (d, *J* = 8.8 Hz, 4H); ^{13}C NMR ($CDCl_3$): *δ* 14.1, 22.7, 25.2, 26.0, 29.1, 29.3, 29.4, 29.6 (for two carbons), 29.8, 31.9, 68.1, 72.8 (t, *J* = 3.6 Hz), 75.2 (t, *J* = 2.1 Hz), 98.6 (t, *J* = 18.3 Hz), 100.6 (t, *J* = 3.6 Hz), 113.8, 114.6, 133.3, 137.7 (tt, *J* = 12.5, 2.9 Hz), 139.6–142.5 (ddt, *J* = 246.4, 13.9, 5.1 Hz), 145.6–148.6 (dm, *J* = 250.1 Hz), 160.0; ^{19}F NMR(CDCl3, C6F6): *δ* –139.62 to –139.73 (m, 4F), –158.86 to –158.98 (m, 4F); IR (KBr): *ν* 2922, 2852, 1604, 1517, 1492, 1291, 1248, 1173, 1021 cm^{-1}; HRMS (FAB) Calcd for (M^+) $C_{54}H_{62}F_8O_4$: 926.4520, Found: 926.4524.

1,8-Bis(4-[2-(4-decyloxyphenyl)ethyn-1-yl]2,3,5,6-tetrafluorophenoxy)octane (2*8*)[3]

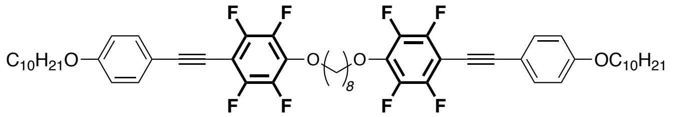

2*8*

Yield: 55%, white solid; M.p.: 100 °C; ^{1}H NMR (CDCl3): *δ* 0.89 (t, *J* = 6.4 Hz, 6H), 1.23–1.55 (m, 34H), 1.79 (quin, *J* = 6.8 Hz, 10H), 3.97 (t, *J* = 6.8 Hz, 4H), 4.25 (t, *J* = 6.4 Hz, 4H), 6.88 (d, *J* = 8.8 Hz, 4H), 7.49 (d, *J* = 8.8 Hz, 4H); ^{13}C NMR ($CDCl_3$): *δ* 14.1, 22.7, 25.4, 26.0, 29.1, 29.2, 29.3, 29.4, 29.5 (for two carbons), 29.8, 31.9, 68.1, 72.9 (t, *J* = 3.6 Hz), 75.4 (t, *J* = 3.0 Hz), 98.5 (t, *J* = 18.3 Hz), 100.6 (t, *J* = 2.9 Hz), 113.8, 114.6, 133.3, 137.8 (tt, *J* = 12.5, 2.9 Hz), 139.7–142.6 (dm, *J* = 247.2 Hz), 145.6–148.6 (dm, *J* = 250.9 Hz), 160.0; ^{19}F NMR(CDCl3, C6F6): *δ* –139.67 to –139.82 (m, 4F), –158.83 to –158.97 (m, 4F); IR (KBr): *ν* 2922, 1604, 1492, 1291, 1248, 1142, 1014, 996 cm^{-1}; HRMS (FAB) Calcd for (M^+) $C_{56}H_{66}F_8O_4$: 954.4833, Found: 954.4823.

1,10-Bis(4-[2-(4-decyloxyphenyl)ethyn-1-yl]2,3,5,6-tetrafluorophenoxy)decane (2*10*)[3]

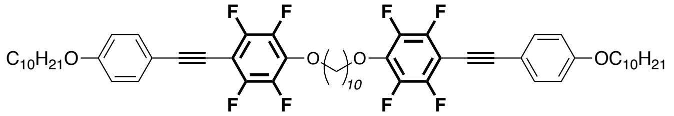

2*10*

Yield; 24%, white solid; M.p.: 97 °C; ^{1}H NMR (CDCl3): *δ* 0.88 (t, *J* = 6.4 Hz, 6H), 1.23–1.40 (m, 30H), 1.40–1.50 (m, 8H), 1.73–1.83 (m, 10H), 3.97 (t, *J* = 6.8 Hz, 4H), 4.25 (t, *J* = 6.4 Hz, 4H), 6.88 (d, *J* = 8.8 Hz, 4H), 7.49 (d, *J* = 8.8 Hz, 4H); ^{13}C NMR ($CDCl_3$): *δ* 14.1, 22.7, 25.5, 26.0, 29.1 (for two carbons), 29.3, 29.4 (for two carbons), 29.5 (for two carbons), 29.9, 31.9, 68.1, 72.9 (t, *J* = 3.6 Hz), 75.4 (t, *J* = 2.2 Hz), 98.4 (t, J = 18.3 Hz), 100.5 (t, *J* = 3.0 Hz), 113.8, 114.6, 133.3, 137.8 (tt, *J* = 11.7, 2.9 Hz), 139.7–142.5 (ddt, *J* = 247.2, 14.0, 4.4 Hz), 145.7–148.6 (dm, *J* = 249.8 Hz), 160.0; ^{19}F NMR(CDCl3, C6F6): *δ* –139.72 to –139.83 (m, 4F), –158.82 to –158.93 (m, 4F); IR (KBr): *ν* 2922, 1605, 1491, 1249, 1142, 1012, 980 cm^{-1}; HRMS (FAB) Calcd for (M^+) C58H70F8O4: 982.5146, Found: 982.5151.

[3] S. Inui, H. Kitaoka, Y. Eguchi, M. Yasui, T. Konno, S. Yamada, *Crystals* 15 (2025): 840.

1,4-Bis[2,3,5,6-tetrafluoro-4-{2-(4-decyloxyphenyl)ethyn-1-yl}benzoyloxy]butane (3_4)[4]

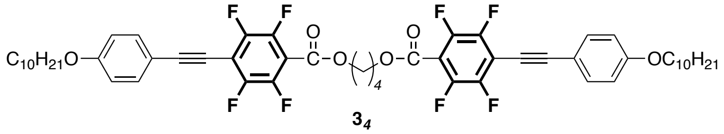

3_4

Yield: 70% (White solid); M.P.: 111 °C (determined by DSC); ^{1}H NMR ($CDCl_3$): δ 0.88 (t, J = 6.4 Hz, 6H), 1.21–1.54 (m, 28H), 1.79 (quin, J = 6.8 Hz, 8H), 3.98 (t, J = 6.4 Hz, 4H), 4.40 (t, J = 6.4 Hz, 4H), 6.89 (d, J = 8.8 Hz, 4H), 7.52 (d, J = 8.8 Hz, 4H); ^{13}C NMR ($CDCl_3$): δ 14.1, 22.7, 25.4, 26.0, 28.3, 29.1, 29.3, 29.4, 29.6, 31.9, 66.5, 68.2, 73.0 (t, J = 3.7 Hz), 104.7 (t, J = 2.9 Hz), 108.1 (tt, J = 2.9 Hz), 111.9 (t, J = 16.2 Hz), 113.0, 114.8, 133.8, 143.1–146.1 (dm, J = 256.8 Hz), 145.1–148.0 (dm, J = 253.0 Hz), 159.6, 160.6; ^{19}F NMR ($CDCl_3$): δ –137.32 to –137.48 (m, 4F), –141.22 to –141.39 (m, 4F); IR (KBr): ν 2953, 2921, 2850, 2213, 1730, 1602, 1515, 1474, 1329, 1295, 1167, 994 cm^{-1}; HRMS: (FAB+) *m/z* [M]$^+$ Calcd for $C_{54}H_{58}F_8O_6$: 954.4106; Found: 954.4109.

1,6-Bis[2,3,5,6-tetrafluoro-4-{2-(4-decyloxyphenyl)ethyn-1-yl}benzoyloxy]hexane (3_6)[4]

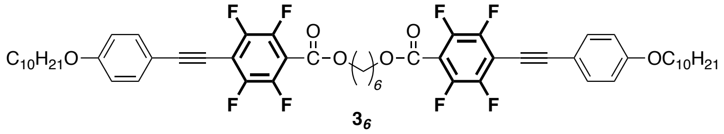

3_6

Yield: 70% (White solid); M.P.: 98 °C (determined by DSC); ^{1}H NMR ($CDCl_3$): δ 0.88 (t, J = 6.4 Hz, 6H), 1.21–1.40 (m, 26H), 1.4–1.55 (m, 6H), 1.79 (quin, J = 6.8 Hz, 8H), 3.98 (t, J = 6.4 Hz, 4H), 4.40 (t, J = 6.4 Hz, 4H), 6.89 (d, J = 8.8 Hz, 4H), 7.52 (d, J = 8.8 Hz, 4H); ^{13}C NMR ($CDCl_3$): δ 14.1, 22.7, 25.4, 26.0, 28.3, 29.1, 29.3, 29.4, 29.5 (for two carbons), 31.9, 66.5, 68.2, 73.0 (t, J = 3.7 Hz), 104.6 (t, J = 2.9 Hz), 108.1 (tt, J = 18.3, 2.9 Hz), 111.9 (t, J = 16.2 Hz), 113.0, 114.7, 133.7, 143.1–146.0 (dm, J = 255.2 Hz), 145.1–148.0 (dm, J = 251.6 Hz), 159.6, 160.6; ^{19}F NMR ($CDCl_3$): δ –137.43 to –137.57 (m, 4F), –141.38 to –141.52 (m, 4F); IR (KBr): ν 2952, 2925, 2853, 2212, 1733, 1603, 1478, 1337, 1254, 1171, 991, 838 cm^{-1}; HRMS: (FAB+) *m/z* [M]$^+$ Calcd for $C_{56}H_{62}F_8O_6$: 982.4419; Found: 982.4416.

1,8-Bis[2,3,5,6-tetrafluoro-4-{2-(4-decyloxyphenyl)ethyn-1-yl}benzoyloxy]octane (3_8)[4]

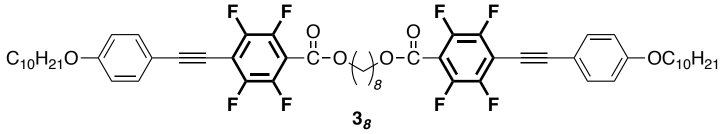

3_8

Yield: 78% (White solid); M.P.: 98 °C (determined by DSC); ^{1}H NMR ($CDCl_3$): δ 0.88 (t, J = 6.4 Hz, 6H), 1.22–1.50 (m, 36H), 1.72–1.85 (m, 8H), 3.98 (t, J = 6.4 Hz, 4H), 4.39 (t, J = 6.4 Hz, 4H), 6.89 (d, J = 8.8 Hz, 4H), 7.52 (d, J = 8.8 Hz, 4H); ^{13}C NMR ($CDCl_3$): δ 14.1, 22.7, 25.7, 26.0, 28.4, 29.0, 29.1, 29.3, 29.4, 29.5 (for two carbons), 31.9, 66.8, 68.2, 73.0 (t, J = 4.4 Hz), 104.6 (t, J = 3.6 Hz), 108.1 (tt, J = 16.8, 3.0 Hz), 111.9 (t, J = 16.1 Hz), 113.0, 114.7, 133.7, 143.1–146.0 (dm, J = 256.0 Hz), 145.1–147.9 (dm, J = 253.0 Hz), 159.6, 160.6; ^{19}F NMR ($CDCl_3$): δ –137.46 to –137.62 (m, 4F), –141.41 to –141.56 (m, 4F); IR (KBr): ν 2932, 2853, 2223, 1734, 1606, 1476, 1469, 1250, 1172, 990, 843 cm^{-1}; HRMS: (FAB+) *m/z* [M]+ Calcd for $C_{58}H_{66}F_8O_6$: 1010.4732; Found: 1010.4725.

[4] S. Inui, Y. Eguchi, M. Morita, M. Yasui, T. Konno, S. Yamada, *Crystals* 15 (2025): 1050.

2,3-Difluoro-4-[2-{4-(7,7,8,8,9,9,10,10,10-nonafluorodecyloxy)phenyl}ethyn-1-yl]benzonitrile (4F6H-4)[5]

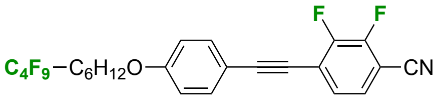

4F6H-4

Yield: 12%; T_m: 82 °C, as determined via DSC; ^{1}H NMR ($CDCl_3$): δ 1.41–1.55 (m, 4H), 1.66 (quin, J = 7.2 Hz, 2H), 1.82 (quin, J = 7.2 Hz, 2H), 1.98–2.16 (m, 2H), 4.00 (t, J = 6.4 Hz, 2H), 6.89 (d, J = 8.8 Hz, 2H), 7.34 (ABq, J = 8.8 Hz, 2H), 7.51 (d, J = 8.8 Hz, 2H); ^{13}C NMR ($CDCl_3$): δ 20.0, 25.7, 28.8, 28.9, 30.7 (t, J = 22.7 Hz), 67.8, 79.4 (d, J = 3.7 Hz), 100.9 (d, J = 4.3 Hz), 101.9 (d, J = 11.8 Hz), 113.3, 114.6, 114.7, 120.2 (d, J = 12.4 Hz), 127.3 (d, J = 4.4 Hz), 128.2 (d, J = 3.7 Hz), 133.2 (d, J = 31.5 Hz), 133.6, 150.4 (dd, J = 272.6, 18.3 Hz), 151.7 (dd, J = 261.8, 14.7 Hz), 160.3, and the four signals that should be assigned to C_4F_9 are split by the F atoms, and their intensities are too low for accurate assignment; ^{19}F NMR ($CDCl_3$, C_6F_6): δ –82.33 (t, J = 9.8 Hz, 3F), –115.94 (quin, J = 16.2 Hz, 2F), –125.80 (q, J = 7.9 Hz, 2F), –127.37 (t, J = 10.9 Hz, 2F), –131.64 (dd, J = 17.7, 5.6 Hz, 1F), –133.25 (dd, J = 17.7, 5.6 Hz, 1F); IR (KBr): ν 2948, 2872, 2242, 2216, 1602, 1514, 1472, 1249, 1172, 1033, 829 cm^{-1}; HRMS (FAB) calc. for [M$^+$] $C_{25}H_{18}F_{11}NO$: 557.1213, found: 557.1213.

2,3-Difluoro-4-[2-{4-(5,5,6,6,7,7,8,8,9,9,10,10,11,11,12,12,12-heptadecafluorododecyloxy)phenyl}ethyn-1-yl]benzonitrile (8F4H-4)[5]

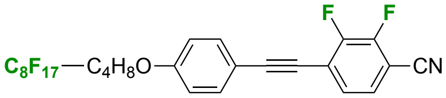

8F4H-4

Yield: 48%; T_m: 108 °C, as determined via DSC; ^{1}H NMR ($CDCl_3$): δ 1.79–1.96 (m, 4H), 2.17 (hept, J = 8.0 Hz, 2H), 4.04 (t, J = 6.0 Hz, 2H), 6.89 (d, J = 8.8 Hz, 2H), 7.31–7.38 (m, 2H), 7.51 (d, J = 8.8 Hz, 2H); ^{13}C NMR ($CDCl_3$): δ 17.3, 25.6, 30.7 (t, J = 21.9 Hz), 67.4, 79.4 (d, J = 4.4 Hz), 100.8 (d, J = 3.7 Hz), 102.0 (d, J = 12.3 Hz), 112.8 (d, J = 3.6 Hz), 113.6, 114.7, 120.2 (d, J = 12.5 Hz), 127.3 (d, J = 4.4 Hz), 128.2 (d, J = 3.7 Hz), 132.7 (d, J = 30.0 Hz), 133.7, 150.4 (dd, J = 255.3, 11.6 Hz), 151.7 (dd, J = 261.8, 14.6 Hz), 160.0, and the eight signals that should be assigned to C_8F_{17} are split by the F atoms, and their intensities are too low for accurate assignment; ^{19}F NMR ($CDCl_3$, C_6F_6): δ –82.01 (t, J = 9.8 Hz, 3F), –115.71 (quin, J = 14.6 Hz, 2F), –122.84 to –123.36 (m, 6F),
–123.86 to –124.12 (m, 2F), –124.68 to –124.88 (m, 2F), –127.26 to –127.48 (m, 2F), –131.61 (dd, J = 17.7, 5.6 Hz, 1F), –133.20 (dd, J = 17.7, 5.6 Hz, 1F); IR (KBr): ν 3071, 2956, 2879, 2241, 2215, 1604, 1515, 1471, 1250, 1210, 1146, 1025, 951, 831 cm^{-1}; HRMS (FAB) calc. for [M$^+$] $C_{27}H_{14}F_{19}NO$: 729.0772, found: 729.0783.

3,5-Difluoro-4-[2-{4-(7,7,8,8,9,9,10,10,10-nonafluorodecyloxy)phenyl}ethyn-1-yl]benzonitrile (4F6H-5)[5]

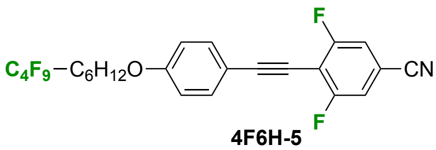

4F6H-5

Yield: 18%; T_m: 93 °C, as determined via DSC; ^{1}H NMR ($CDCl_3$): δ 1.38–1.52 (m, 4H), 1.59 (quin, J = 8.0 Hz, 2H), 1.76 (quin, J = 6.8 Hz, 2H), 1.92–2.10 (m, 2H), 3.93 (t, J = 6.4 Hz, 2H), 6.83 (d, J = 8.8 Hz, 2H), 7.18 (d, J = 6.4 Hz, 2H), 7.46 (d, J = 8.8 Hz, 2H); ^{13}C NMR ($CDCl_3$): δ 20.0, 25.7, 28.8, 28.9, 30.7 (t, J = 22.7 Hz), 67.7, 73.8, 104.2 (t, J = 3.0 Hz), 111.6 (t, J = 11.8 Hz), 114.56, 114.64, 115.2 (d, J = 27.9 Hz), 133.7, 134.0, 159.7, 160.3, 162.4 (dd, J = 255.9, 5.8 Hz), and the four signals that should be assigned to C_4F_9 are split by the F atoms, and their intensities are too low for

[5] S. Yamada, K. Yoshida, M. Hara, T. Agou, M. Yasui, T. Konno, *Journal of Molecular Liquids* 393 (2024): 1235445.

accurate assignment; ^{19}F NMR ($CDCl_3$, C_6F_6): δ –82.34 (t, J = 9.4 Hz, 3F), –105.53 (d, J = 5.6 Hz, 2F), –115.95 (quin, J = 15.1 Hz, 2F), –125.72 to –125.88 (m, 2F), –127.37 (t, J = 12.0 Hz, 2F); IR (KBr): ν 2948, 2871, 2239, 2220, 1602, 1427, 1249, 1131, 1038, 828 cm^{-1}; HRMS (FAB) calc. for [M^+] $C_{25}H_{18}F_{11}NO$: 557.1213, found: 557.1213.

3,5-Difluoro-4-[2-{4-(5,5,6,6,7,7,8,8,9,9,10,10,11,11,12,12,12-heptadecafluorododecyloxy)phenyl}ethyn-1-yl]benzonitrile (8F4H-5)[5]

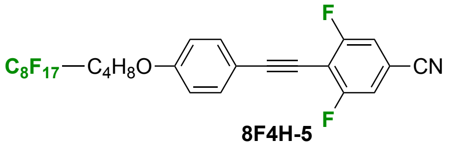

8F4H-5

Yield: 11%; T_m: 124 °C, as determined via DSC; ^{1}H NMR ($CDCl_3$): δ 1.78–1.96 (m, 4H), 2.09–2.26 (m, 2H), 4.04 (t, J = 5.6 Hz, 2H), 6.90 (d, J = 8.8 Hz, 2H), 7.24 (d, J = 6.4 Hz, 2H), 7.53 (d, J = 8.8 Hz, 2H); ^{13}C NMR ($CDCl_3$): δ 17.3, 28.6, 30.7 (t, J = 22.0 Hz), 67.4, 73.9, 104.1 (t, J = 3.7 Hz), 111.7 (t, J = 12.4 Hz), 113.7, 114.7, 115.2 (d, J = 27.7 Hz), 133.7, 134.5, 160.0, 161.8, 162.4 (dd, J = 256.6, 5.2 Hz), and the eight signals that should be assigned to C_8F_{17} are split by the F atoms, and their intensities are too low for accurate assignment; ^{19}F NMR ($CDCl_3$, C_6F_6): δ –82.03 (t, J = 9.4 Hz, 3F), –105.49 (d, J = 5.3 Hz, 2F), –115.62 to –115.83 (m, 2F), –122.9 to –123.4 (m, 6F), –123.8 to –124.2 (m, 2F), –124.6 to –124.9 (m, 2F), –127.3 to –127.6 (m, 2F); IR (KBr): ν 3071, 2958, 2880, 2238, 2219, 1602, 1519, 1426, 1174, 1146, 1038, 870 cm^{-1}; HRMS (FAB) calc. for [M^+] $C_{27}H_{14}F_{19}NO$: 729.0772, found: 729.0773.

N-[4-{2-(4-Cyano-2,6-difluorophenyl)ethyn-1-yl}phenoxy]decyl-*N*-methylimidazolium chloride (6-Cl)[6]

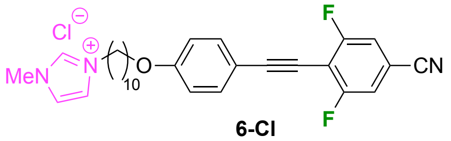

6-Cl

Yield: 59 % (blue solid); T_m: 139 °C; ^{1}H NMR ($CDCl_3$): δ 1.24–1.39 (m, 10H), 1.45 (quin, J = 7.2 Hz, 2H), 1.79 (quin, J = 6.4 Hz, 2H), 1.91 (quin, J = 5.6 Hz, 2H), 3.98 (t, J = 6.8 Hz, 2H), 4.12 (s, 3H), 4.31 (t, J = 7.6 Hz, 2H), 6.89 (d, J = 8.8 Hz, 2H), 7.16 (d, J = 5.2 Hz, 2H), 7.24 (d, J = 6.0 Hz, 2H), 7.52 (d, J = 8.8 Hz, 2H), 11.12 (brs, 1H); ^{13}C NMR ($CDCl_3$): δ 25.9, 26.2, 28.9, 29.0, 29.2 (two alkylene carbons), 29.3, 30.2, 36.6, 50.1, 68.1, 73.8, 104.2 (t, J = 2.9 Hz), 108.8 (t, J = 19.8 Hz), 111.6 (t, J = 12.0 Hz), 113.2, 114.7, 115.1 (d, J = 28.6 Hz), 116.5, 121.4, 123.1, 133.6, 138.6, 160.4, 162.3 (dd, J = 255.9, 5.8 Hz); ^{19}F NMR ($CDCl_3$, C_6F_6): δ –105.54 (d, J = 6.0 Hz, 2F); IR (KBr): ν 3419, 3079, 2924, 2854, 2218, 1607, 1521, 1426, 1037, 832 cm^{-1}; HRMS (FAB) Calcd for (M^+) $C_{29}H_{32}F_2N_3O$: 476.2508, Found: 476.2510.

N-[4-{2-(4-Cyano-2,6-difluorophenyl)ethyn-1-yl}phenoxy]decyl-*N*-methylimidazolium bromide (6-Br)[6]

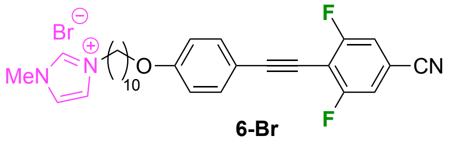

6-Br

Yield: Quant. (yellow solid); T_m: 142 °C; $T_d^{5\,\%}$ = 272 °C; ^{1}H NMR ($CDCl_3$): δ 1.25–1.39 (m, 10H), 1.45 (quin, J = 8.4 Hz, 2H), 1.79 (quin, J = 7.2 Hz, 2H), 1.92 (quin, J = 6.8 Hz, 2H), 3.98 (t, J = 6.4 Hz, 2H), 4.12 (s, 3H), 4.32 (t, J = 7.6 Hz, 2H), 6.89 (d, J = 8.8 Hz, 2H), 7.18 (dt, J = 8.0, 1.6 Hz, 2H), 7.24 (d, J = 6.0 Hz, 2H), 7.52 (d, J = 8.8 Hz, 2H), 10.92 (brs, 1H); ^{13}C NMR ($CDCl_3$): δ 25.9, 26.2, 28.9, 29.0, 29.2, 29.3, 30.2, 36.8, 50.1, 68.1, 73.8, 104.2, 108.8 (t, J = 19.8 Hz), 111.5 (t, J = 11.8 Hz), 113.2, 114.7, 115.1 (d, J = 28.5 Hz), 116.5, 121.5, 123.2, 127.1, 133.6, 138.2, 160.4, 162.3 (dd, J = 256.8, 5.8 Hz); ^{19}F NMR ($CDCl_3$, C_6F_6): δ –105.54 (d, J = 6.0 Hz, 2F); IR (KBr): ν 3423, 3059, 2922, 2850, 2235, 1601, 1514, 1467, 1247, 1178, 836 cm^{-1}; HRMS (FAB) Calcd for (M^+) $C_{29}H_{32}F_2N_3O$: 476.2508, Found: 476.2510.

N-[4-{2-(4-Cyano-2,6-difluorophenyl)ethyn-1-yl}phenoxy]decyl-*N*-methylimidazolium tetrafluoroborate (6-BF_4)[6]

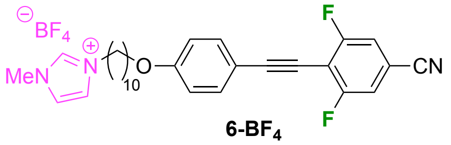

6-BF_4

Yield: 54 % (pale blue solid); T_m: 93 °C; $T_d^{5\,\%}$ = 253 °C; ^{1}H NMR($CDCl_3$): δ 1.26–1.39 (m, 10H), 1.45 (quin, J = 6.4 Hz, 2H), 1.78 (quin, J = 8.0 Hz, 2H), 1.89 (quin, J = 6.4 Hz), 3.98 (t, J = 6.4 Hz, 2H), 4.01 (s, 3H), 4.21 (t, J = 7.2 Hz, 2H), 6.89 (d, J = 8.8 Hz, 2H), 7.19–7.28 (m, 4H), 7.51 (d, J = 8.8 Hz, 2H), 9.33 (brs, 1H); ^{13}C NMR ($CDCl_3$): δ 25.8, 26.0, 28.8, 28.9, 29.1, 29.2, 29.9, 45.1, 49.9, 68.0, 73.7, 104.1, 108.6 (t, J = 19.8 Hz), 111.4 (t, J = 11.0 Hz), 113.0, 114.6, 115.1 (d, J = 28.7 Hz), 116.4, 122.0, 123.6 (one aromatic carbon overlapped with this signal), 133.5, 136.3, 160.3, 162.2 (dd, J = 255.9, 5.8 Hz); ^{19}F NMR ($CDCl_3$, C_6F_6): δ – 105.54 (d, J = 5.6 Hz, 2F); −153.11 (s, 4F); IR (KBr): ν 3412, 3081, 2926, 2855, 2218, 1606, 1426, 1172, 1036, 832 cm^{-1}; HRMS (FAB) Calcd for (M^+) $C_{29}H_{32}F_2N_3O$: 476.2508, Found: 476.2510; Anal. calcd for $C_{29}H_{32}BF_6N_3O$: C, 61.82; H, 5.73; N, 7.46. Found: C, 61.29; H, 5.29, N, 7.34.

---

[6] S. Yamada, Y. Eguchi, T. Yasuda, K. Yoshida, M. Hara, M. Yasui, T. Konno, *Journal of Molecular Liquids* 408 (2024): 125361.

N-[4-{2-(4-cyano-2,6-difluorophenyl)ethyn-1-yl}phenoxy]decyl-N-methylimidazolium hexafluorophosphate (6-PF_6)[6]

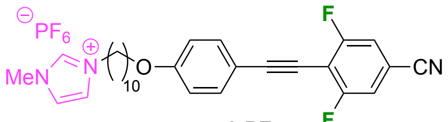

6-PF_4

Yield: 84 % (white solid); T_m: 74 °C; $T_d^{5\,\%}$ = 275 °C; ^{1}H NMR ($CDCl_3$): δ 1.22–1.38 (m, 10H), 1.44 (quin, J = 7.2 Hz, 2H), 1.78 (quin, J = 7.2 Hz, 2H), 1.86 (quin, J = 6.8 Hz, 2H), 3.93 (s, 3H), 3.97 (t, J = 6.4 Hz, 2H), 4.14 (t, J = 7.6 Hz, 2H), 6.89 (d, J = 8.8 Hz, 2H), 7.23 (d, J = 6.4 Hz, 2H), 7.50 (d, J = 8.8 Hz, 2H), 8.62 (brs, 1H); ^{13}C NMR ($CDCl_3$): δ 25.9, 26.1, 28.8, 29.1, 29.16, 29.19, 29.3, 29.9, 36.3, 50.2, 68.1, 73.8, 104.3 (t, J = 2.9 Hz), 108.8 (t, J = 19.8 Hz), 111.6 (t, J = 10.9 Hz), 113.2, 114.7, 115.2 (d, J = 28.5 Hz), 116.5, 121.9, 123.4, 133.6, 136.2, 160.4, 162.3 (dd, J = 255.9, 5.8 Hz); ^{19}F NMR ($CDCl_3$, C_6F_6): δ –73.89 (d, $^1J_{FP}$ = 712 Hz, 6F), −105.55 (d, J = 6.4 Hz, 2F); IR (KBr): ν 3107, 3122, 2926, 2854, 2218, 1605, 1520, 1427, 1264, 1170, 1038, 971, 839 cm^{-1}; HRMS (FAB) Calcd for (M^+) $C_{29}H_{32}F_2N_3O$: 476.2508, Found: 476.2519; Anal. calcd for $C_{29}H_{32}F_9N_3OP$: C, 56.04; H, 5.19; N, 6.76. Found: C, 55.65; H, 5.30, N, 6.27.

N-[4-{2-(4-Cyano-2,6-difluorophenyl)ethyn-1-yl}phenoxy]decyl-N-methylimidazolium triflate (6-OTf)[6]

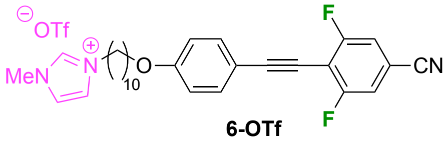

6-OTf

Yield: Quant. (white solid); T_m: 36 °C; $T_d^{5\,\%}$ = 378 °C; ^{1}H NMR ($CDCl_3$): δ 1.24–1.38 (m, 10H), 1.43 (quin, J = 7.2 Hz, 2H), 1.77 (quin, J = 7.6 Hz, 2H), 1.86 (quin, J = 7.6 Hz, 2H), 3.91–4.02 (m, 5H), 4.18 (t, J = 7.6 Hz, 2H), 6.88 (d, J = 8.4 Hz, 2H), 7.23 (d, J = 5.6 Hz, 2H), 7.32 (d, J = 23.6 Hz, 2H), 7.50 (d, J = 8.4 Hz, 2H), 9.14 (brs, 1H); ^{13}C NMR (MeOH-d_4): δ 27.0, 27.2, 30.0, 30.2, 30.3, 30.5, 31.1, 36.4, 50.8, 69.2, 74.4, 104.9, 109.3 (t, J = 20.5 Hz), 113.2 (t, J = 11.7 Hz), 114.4, 115.9, 116.7 (d, J = 29.3 Hz), 120.2, 123.3, 123.6, 124.9, 134.6, 137.8, 162.0, 163.6 (dd, J = 254.5, 5.9 Hz); ^{19}F NMR ($CDCl_3$, C_6F_6): δ –105.50 (d, J = 5.6 Hz, 2F), −79.72 (s, 3F); IR (KBr): ν 3602, 3055, 2924, 2856, 2216, 1604, 1281, 1256, 1171, 1029, 831 cm^{-1}; HRMS (FAB) Calcd for (M^+) $C_{29}H_{32}F_2N_3O$: 476.2508, Found: 476.2510; Anal. calcd for $C_{30}H_{32}F_5N_3O_4S$: C, 57.59; H, 5.16; N, 6.72. Found: C, 57.32; H, 5.03, N, 6.03.

N-[4-{2-(4-cyano-2,6-difluorophenyl)ethyn-1-yl}phenoxy]decyl-N-methylimidazolium bis(trifluoromethane)sulfoimide (6-NTf_2)[6]

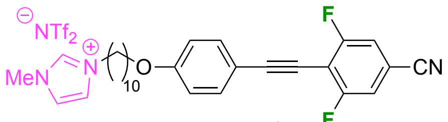

6-NTf_2

Yield: 96% (white solid); T_m: 63 °C determined by DSC during the 2nd heating process; $T_d^{5\%}$ = 354 °C; ^{1}H NMR ($CDCl_3$): d 1.25–1.40 (m, 10H), 1.45 (quin, J = 5.6 Hz, 2H), 1.79 (quin, J = 6.8 Hz, 2H), 1.94 (quin, J = 6.8 Hz, 2H), 3.98 (t, J = 6.4 Hz, 2H), 4.11 (s, 3H), 4.31 (t, J = 7.6 Hz, 2H), 6.89 (d, J = 8.8 Hz, 2H), 7.20–7.25 (m, 4H), 7.52 (d, J = 8.8 Hz, 2H), 10.34 (brs, 1H); ^{13}C NMR ($CDCl_3$): d 25.9, 26.2, 28.9, 29.1, 29.2 (two alkylene carbons), 29.3, 30.2, 37.0, 50.4, 68.1, 73.8, 104.3, 108.8 (t, J = 19.8 Hz), 111.6 (t, J = 11.7 Hz), 113.3, 114.7, 115.2 (d, J = 28.6 Hz), 116.5, 121.5, 123.0, 133.7, 137.7, 160.4, 162.4 (dd, J = 256.8, 5.8 Hz); ^{19}F NMR ($CDCl_3$, C_6F_6): d –80.19 (s, 6F), –105.5d (d, J = 5.6 Hz, 2F); IR (KBr): n 3153, 3095, 2929, 2857, 2217, 1601, 1518, 1428, 1348, 1193, 1037, 863 cm^{-1}; HRMS (FAB) Calcd for (M^+) $C_{29}H_{32}F_2N_3O$: 476.2508, Found: 476.2519; Anal. calcd for $C_{31}H_{32}F_8N_4O_5S_2$: C, 49.20; H, 4.26; N, 7.40. Found: C, 49.39; H, 4.27, N, 7.25.
